# A ferroptosis-related prognostic model with excellent clinical performance based on the exploration of the mechanism of oral squamous cell carcinoma progression

**DOI:** 10.1038/s41598-023-27676-3

**Published:** 2023-01-26

**Authors:** Xin Fan, Yun Zhong, Fang Yuan, Lingling Zhang, Ying Cai, Lan Liao

**Affiliations:** 1 The Affiliated Stomatological Hospital of Nanchang University, Nanchang, Jiangxi Province China; 2 The Key Laboratory of Oral Biomedicine, Nanchang, Jiangxi Province China; 3 Jiangxi Province Clinical Research Center for Oral Diseases, Nanchang, Jiangxi Province China; 4 The Affiliated Hospital of Jinggangshan University, Jian, Jiangxi Province China; 5 Department of Colorectal Surgery, The Sixth Affiliated Hospital, Sun Yat-sen University, Guangzhou, Guangdong Province China; 6 Ophthalmology and Otorhinolaryngology, Fenyi County people′s Hospital, Xinyu, Jiangxi Province China; 7 The Stomatology College of Nanchang University, Nanchang, Jiangxi Province China

**Keywords:** Cancer, Computational biology and bioinformatics

## Abstract

As a hot topic today, ferroptosis is closely involved in the progression and treatment of cancer. Accordingly, we built a prognostic model around ferroptosis to predict the overall survival of OSCC patients. We used up to 6 datasets from 3 different databases to ensure the credibility of the model. Then, through differentially expressed, Univariate Cox, and Lasso regression analyses, a model composed of nine prognostic-related differently expressed ferroptosis-related genes (CISD2, DDIT4, CA9, ALOX15, ATG5, BECN1, BNIP3, PRDX5 and MAP1LC3A) were constructed. Moreover, Kaplan–Meier curves, Receiver Operating Characteristic curves and principal component analysis used to verify the model's predictive ability showed the model's superiority. To deeply understand the mechanism of ferroptosis affecting the occurrence, development and prognosis of OSCC, we performed enrichment analysis in different risk groups identified by the model. The results showed that numerous TP53-related, immune-related and ferroptosis-related functions and pathways were enriched. Further immune microenvironment analysis and mutation analysis have once again revealed the correlation between risk score and immunity and TP53 mutation. Finally, the correlation between risk score and OSCC clinical treatment, as well as Nomogram show the brilliant clinical application prospects of the prognostic model.

## Introduction

Oral squamous cell carcinoma (OSCC) accounts for more than 95% of oral cancers^1^. In 2020, there were more than 370,000 new cases of oral cancer (2.0%), and the death toll surpassed 170,000 (1.8%)^2^. In the past few decades, the number of young people suffering from OSCC has sharply increased year by year^3^. Alcohol abuse, smoking, betel nut chewing, and human papillomavirus (HPV) infection are the most common risk factors that lead to OSCC^4^. At present, surgery is the mainstay curative treatment for OSCC. The patient's clinical and pathological characteristics determine whether to perform the adjuvant treatment (radiotherapy or chemotherapy)^5^. If early OSCC can be detected in time and treated appropriately, the 5-year survival rate can reach 90%^6^. Regrettably, most oral cancers are discovered until the later stages, resulting in a poor prognosis for OSCC^7^, and more than half of the affected patients with OS (Overall Survival) of less than 5 years^8^. Even though we have made progress in surgery and adjuvant chemotherapy because patients often have a regional recurrence and lymph node metastasis, the later prognosis is still poor^3^. In recent years, immune checkpoint inhibitors (ICIs) have been approved for OSCC, but not all patients can benefit from ICIs^9^. Hence, as the limitations of various treatments, combined with OSCC's intense aggressiveness and frequent metastasis and recurrence^10^, it is necessary to find a model that can be used to predict the prognosis and treatment effects of OSCC.

Ferroptosis is a newly recognized non-apoptotic programmed cell death process with unique morphological and biodynamic characteristics. Lipid peroxidation accumulation and iron dependence are the two main characteristics of ferroptosis^11^. Ferroptosis can be regulated by a gamut of small molecules, multiple signaling pathways, and organelles, inducing cancer cell death^12^. These facts confirm the possibility of cancer treatment with ferroptosis. More and more studies have demonstrated that ferroptosis plays an essential role in treating and prognosis of numerous diseases in recent years. Studies have shown that ferroptosis is closely related to cancer immunotherapy and tumor-infiltrating immune cells^13^. Lang et al. also found that radiation can induce tumor ferroptosis, indicating that radiation therapy can also achieve anti-tumor through ferroptosis Efficacy^14^. Collectively, ferroptosis plays a vital role in the oncogenesis and treatment of tumors and provides a new option for cancer prognosis prediction^15^.

ICIs have an excellent therapeutic effect on some patients with metastatic cancer, but they lack predictive biomarkers. Tumor mutation burden (TMB), defined as the total number of somatic/acquired mutations per coding area of a tumor genome (Mut/Mb) 22, has been demonstrated by many studies as a predictive biomarker potential for potential the identification of patients with cancer^16,17^. Tumor protein 53 (TP53) is the first tumor suppressor gene to be identified. Its mutations are related to the initiation and progression of various human cancers^18^. Studies have revealed TP53 can inhibit tumor function by inducing ferroptosis^19^. The above studies suggested that we may explain the relationship between ferroptosis and the prognosis of OSCC from the perspectives of ICIs, TMB, TP53 mutation.

Taken together, the various treatment methods for OSCC have their shortcomings, and there is the absence of appropriate models to predict prognostic. To remedy the problems, we used prognostic-related differentially expressed ferroptosis-related genes (PR-DE-FRGs) obtained from five cohorts of the TCGA, GEO, and ICGC databases to construct a prognostic model and used 6 datasets from 3 databases to verify the model. We then explored the mechanism of ferroptosis in the initiation, progression, and prognosis of OSCC from multiple perspectives such as multiple signaling pathways, immunity, gene mutations, and cell stemness characteristics. Given the importance of ICIs to immunotherapy, we further explored the correlation between the risk score and the expression of ICIs-related and M6A-related genes. Finally, considering the relevance of ferroptosis and clinical treatment, we ran the correlation analysis between immunophenoscore (IPS) and the expression of 9 PR-DE-FRGs and between the expression of 9 PR-DE-FRGs and chemotherapeutics. And constructed a nomogram to predict the prognosis of the patient.

## Materials and methods

### DE-FRGs data collection and analysis

**Figure 1:**
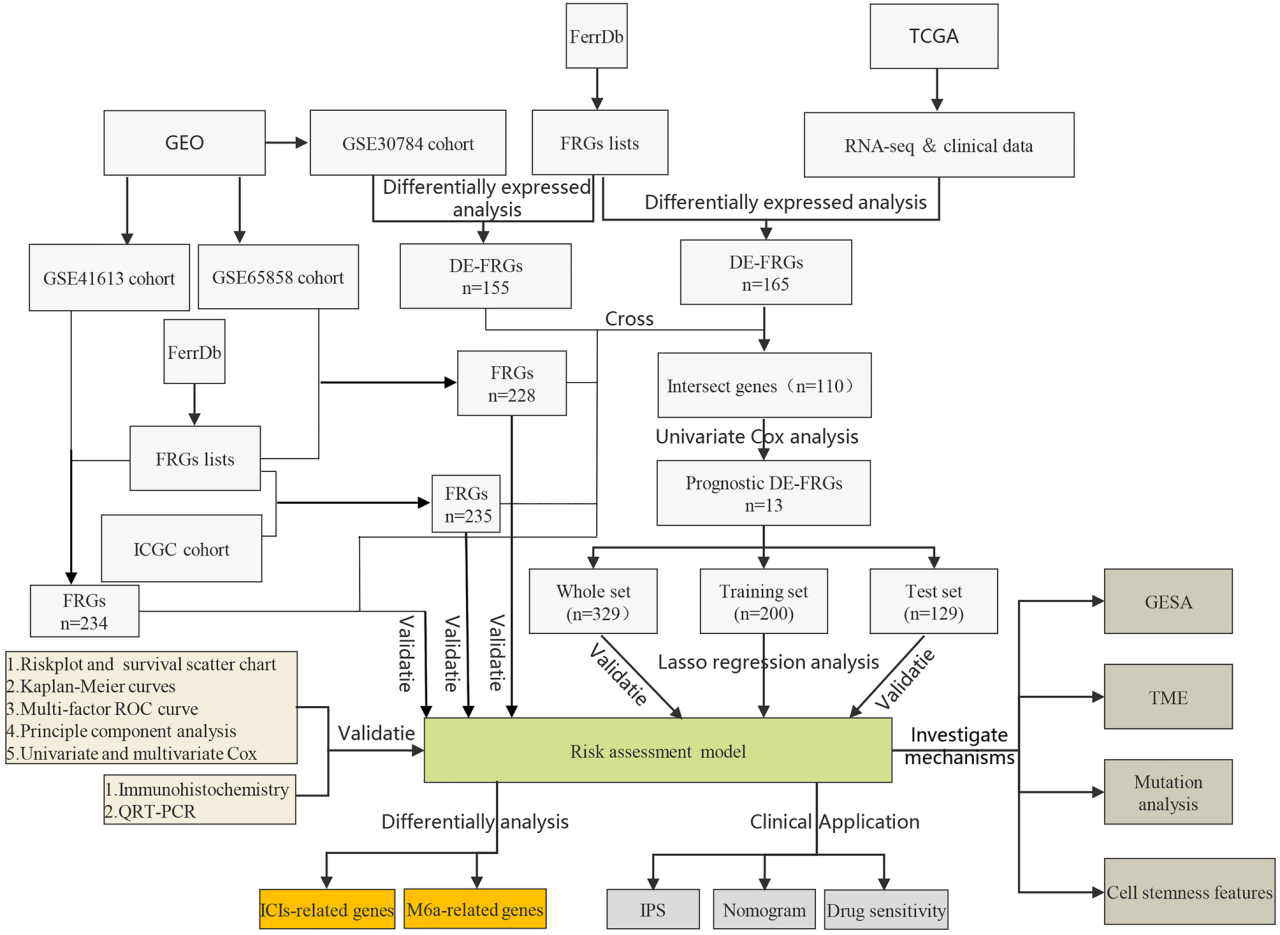
The workflow of the whole study.

All methods of this study were performed in accordance with relevant guidelines and regulations. The workflow of this research is shown in Fig. 1. A total of 5 OSCC cohorts were applied in this study. First, on February 9, 2021, OSCC samples originating in oral cavity (tongue, lips, cheek, palate, gums, floor of the mouth, etc.) were downloaded from the TCGA database (http://cancergenome.nih.gov/) of head and neck squamous cell carcinoma (TCGA-HNSCC) Sample. And we extracted RNA sequencing of 363 samples of OSCC (331 cases of OSCC and 32 cases of adjacent normal tissues) and clinical data of 346 samples. At the same time, somatic gene mutation data has also been downloaded. Thereafter, on July 8, 2021, the RNA sequencing data of 229 samples (167 OSCC, 17 abnormal hyperplasia, and 45 normal oral tissues) of the GSE30784 cohort, the RNA sequencing and clinical data of 97 OSCC samples from the GSE41613 cohort and another RNA sequencing and clinical data of 83 OSCC samples from the GSE65858 cohort. The above three external validation data cohorts were acquired from the GEO database (https://www.ncbi.nlm.nih.gov/geo/). Not only that, on July 14, 2021, the RNA sequencing and clinical data of 28 oral cancer samples were downloaded from the ICGC website (https://dcc.icgc.org/releases/current/Projects/ORCA-IN). The last database was the FerrDb database (http://www.zhounan.org/ferrdb), which has 259 FRGs from the 784 articles on ferroptosis in the PubMed database. It is the source of the list of FRGs that we required for our research. Because these data are publicly available in TCGA, GEO, and ICGC databases, this study didn't require the approval of the local ethics committee. Extracted the gene expression quantification of the same FRGs between the list of FRGs and RNA sequencing data from 5 cohorts (including 246 FRGs in the TCGA cohort, 234 FRGs in the GSE30784 cohort, 234 FRGs in the GSE41613 cohort, 228 FRGs in the GSE65858 cohort, and 235 FRGs in the ICGC-ORCA-IN cohort). In addition, we used the R package “limma” to perform differential expression analysis on OSCC samples and adjacent normal tissues from TCGA, with a false discovery rate (FDR) cut-off < 0.05, to obtain differentially expressed ferroptosis-related genes (DE-FRGs). A similar method was used to obtain DE-FRGs using RNA sequencing data of cancer, abnormal hyperplasia, and normal oral tissue in the GSE30784 cohort. Finally, the R “venn” package determined the overlapping genes among DE-FRGs obtained from the TCGA and GSE30784 cohort and FRGs obtained from the other three cohorts.

### Establishment of regulatory network and prognostic model

We collated the data of 5 cohorts to obtain samples with complete OS and RNA expression data. A univariate COX analysis was performed with a p-value cut-off of 0.05 to screen out prognosis related DE-FRGs (PR-DE-FRGs). The integrated samples from TCGA were randomly divided into the training set (n = 200) and the test set (n = 129) at a 6:4 ratio. The 13 PR-DE-FRGs from the training set were used to construct a lasso regression analysis to obtain highly relevant genes, thereby further improving the accuracy of predicting the clinical prognosis of OSCC patients. Finally, a prognostic model based on 9 PR-DE-FRGs was constructed by choosing the best Lasso penalization parameter (λ) determined by the smallest k-fold cross-validation with K = 10 (Supplementary Fig. S1A,B). Following this, the coefficients obtained by the lasso regression algorithm were used in the risk score equation:

$${\text{Risk\, score}} = \sum (Gene\,expression\,value{\text{s}} \times { }Gene\,corresponding\,coefficient)$$

In the STRING database, a protein–protein interaction network (PPI) composed of 13 PR-DE-FRGs was built with the minimum required interaction score was set to confidence score (0.150). The correlation analysis of 13 genes and the visualization of related networks are realized using R packages "reshape2", "psych", "RColorBrewer" and "igraph". In addition, we also divided all samples into high expression group and low expression group according to the best cutoff value of each PR-DE-FRGs expression value. Next, Kaplan–Meier survival curve was used to analyze the differences in survival between samples of high expression group and low expression group of these PR-DE-FRGs.

### Evaluations of the prognostic model’s predictive ability

To better evaluate the prognostic model's predictive ability, we used the training, test, and whole sets of TCGA, the GSE41613 and the GSE65858 sets of GEO, and the ORCA-IN set of ICGC at the same time. First, we divided the dataset samples into high-risk and low-risk groups based on the median value of the risk score of each case in the training, test, and whole sets respectively. We used the R packages "survival" and "survminer" to verify the performance of the Kaplan–Meier method to distinguish the OS of OSCC patients. Besides that, the Receiver Operating Characteristic (ROC) curves established based on six datasets were used to evaluate the model's accuracy in predicting prognosis. At the same time, the multivariate ROC curves established by the three sets of TCGA were used to verify the optimality of the model in predicting prognosis compared with other factors. Then, the "prcomp" function of the R package "stats" was used for the principal component analysis (PCA) of the expression of each group's genes. Finally, to further evaluate whether the risk score can be used as an independent prognostic indicator, we performed univariate and multivariate Cox regression analysis on the three sets of TCGA factors (age, gender, grade, stage, T, N, and risk score).

### Immunohistochemistry

We used the results of immunohistochemistry (IHC) staining from the human protein atlas database (https://www.proteinatlas.org/) to further confirm the differential expression of 9 modeled genes between OSCC tissue and normal oral tissue. Finally, the IHC images of 7 PR-DE-FRGs protein expression in OSCC and normal oral tissues were obtained.

### Quantitative Real-time PCR (QRT-PCR)

Unfortunately, we were unable to verify the differential expression of BNIP3, DDIT4 and MAP1LC3A between OSCC and normal tissues by IHC staining images. Therefore, we hoped to validate the differential expression of these three genes at the mRNA level between OSCC and normal tissues by QRT-PCR experiment. Twenty-four pairs of matched OSCC tissues and adjacent normal oral tissues were obtained from patients undergoing surgical treatment at the Second Affiliated Hospital of Nanchang University and Affiliated Stomatological Hospital of Nanchang University. This work was not only approved by the Ethics Committee of the Affiliated Stomatological Hospital of Nanchang University (2021-08-015), but also obtained the written informed consent of all patients.

Sangon Biotech provided primers for detection genes for us (Supplementary Table 2). Total RNA extracted from tissues using the TransZol Up Plus RNA Kit (TRANS, Beijing, China) was reverse transcribed into cDNA using EasyScript First-Strand cDNA Synthesis SuperMix (TRANS, Beijing, China). The mRNA expression levels of BNIP3, DDIT4 and MAP1LC3A were amplified and detected using the Archimed quantitative PCR detection system and PerfectStart® Green qPCR SuperMix (TRANS, Beijing, China).

We normalized all detected values to relative expression values of β-actin using the 2^−ΔΔCt^ method. Paired t-test was run to compare the relative mRNA expression value of these 3 genes between 24 pairs of matched OSCC and adjacent normal oral tissues.

### Stratified analyses

First, we used heatmaps to visualize the distribution of clinical characteristics in the high-risk and low-risk groups from TCGA whole set. Subsequently, we used the Wilcoxon signed-rank test and boxplots to visualize differences in risk score between different subgroups of these clinical features. We also compared the expression differences of the 9 modeled genes between different grades/stages. Finally, the Kaplan–Meier method was used to assess the power of risk score to predict OS in subgroups with different clinical characteristics.

### Gene set enrichment analysis

The GSEA software (V4.1.0) was used to perform Gene Ontology (GO) analysis and Kyoto Encyclopedia of Genes and Genomes (KEGG) enrichment analysis based on risk group and gene expression matrix data. "c2.cp.kegg.v7.4.symbols.gmt" and "c5.go.v7.4.symbols.gmt" were selected as the gene set database. Normalized enrichment score (NES) was calculated by setting the permutation value to 1000, and significant enrichment pathways and functions were screened using a P-value < 0.05^20^.

### Immune microenvironment analysis

The R package "estimate" was used to calculate each sample's immune score and stromal score. To quantify the score of 16 immune cells and 13 immune functions, we used a single-sample gene set enrichment analysis (ssGSEA) based on the R packages "GSEABase" and "gsva". We not only compared the differences between the high-risk and the low-risk group in the immune score and stromal score, but also analyzed the correlation between risk score and immune score or stromal score using Spearman correlation test. Then a heatmap was used to display the distribution differences of 16 immune cells and 13 immune functions of each sample with a different risk score. The Spearman correlation test was again used to evaluate the relevance between the risk score and each immune cell or immune function score. Finally, we drew boxplots to show the differences in immune cells and immune functions score between different risk groups.

### Mutational signatures

In the enrichment analysis, the high-risk group is enriched for many P53-related biological processes. We therefore further performed a TP53 mutation-related analysis. Somatic gene mutations in OSCC samples and corresponding clinical data were downloaded from TCGA database and then used to evaluate the relationship between mutations and prognostic models. We first used VarScan to detect the MAF files of somatic mutations^21^ and then the R package "GenVisR" was used to visualize the 20 most frequently mutated genes in different risk groups. The R package "maftools" was used for calculating TMB, and Kaplan–Meier methods were used to compare the OS difference between the high TMB and low TMB group to explore the influence of TMB on the prognosis of patients. According to the mutation status of TP53, TCGA samples were divided into wild and mutation group. Then the difference in risk score between the two groups was analyzed to show the correlation between TP53 mutation and ferroptosis in OSCC. Kaplan–Meier method was performed to compare the OS difference between the TP53 mutant and the wild group. Based on RNA sequencing, to further explore the relationship between TP53 mutation status and immune infiltrating cells, the deconvolution algorithm CIBERSORT^22^ was performed with p-value < 0.05, and finally obtained proportion matrix data of 22 immune cells in each tumor sample. The R package "Limma" was used to compare the differences in the content of infiltrating immune cells between the two groups, and the R package "Vioplot" was used to visualize the results.

### The stem-cell characteristics of OSCC

We downloaded the accurately calculated tumor cell stemness index (mDNAsi and mRNAsi) from related research. The stemness index in the research was calculated based on the OCLR algorithm trained on the types of stem cells (ESCs, embryonic stem cells; iPSCs, induced pluripotent stem cells) and their differentiated ectoderm, mesoderm and endoderm progenitor cells^23^. OCLR-based transcriptomic and epigenetic signatures were applied to TCGA datasets to calculate the mRNAsi and mDNAsi^23^. Each mDNAsi/mRNAsi ranged from low (zero) to high (one)^23^. We analyzed the difference of mRNAsi /mRNAs between OSCC and adjacent normal tissues samples. Then, Spearman correlation analysis was used to determine the correlation between risk score and mRNAsi /mRNAsi, and Wilcoxon rank-sum test was used to compare the difference of mRNAsi/mRNAsi between high-risk and low-risk groups. Finally, the Wilcoxon rank-sum test was performed to gain insight into the differences in mRNAsi /mRNAsi between different subgroups of each clinicopathological feature.

### Correlation analysis between risk score and ICIs or M6A related gene expression values

Given that immune check-point inhibitors (ICIs) play a significant role in immunotherapy, we performed the Spearman correlation test to explore the correlation between the risk score and the expression of ICIs-related genes. In addition, to verify the correlation results, we also compared the differences in ICIs-related gene expression between the high-risk and low-risk groups. Similar methods have also been applied to explore the correlation between M6A-related gene expression and prognostic models.

### The clinical application prospects of prognostic models

The IPS of OSCC patients was available through The Cancer Immunome Atlas (TCIA) database (https://tcia.at/home). The patient's IPS was obtained by evaluating the gene expression of the four cell types (effector cells, immunosuppressive cells, MHC molecules, and immunomodulators) that determine immunogenicity^24^. The immunogenicity increases as the IPS score increases^24^. Spearman correlation analysis was used to evaluate the correlation between the expression of 4 types of IPS and 9 types of PR-DE-FRGs to explore the the potential of these PR-DE-FRGs to predict the efficacy of immunotherapy. The NCI-60 database containing 60 different cancer cell lines from 9 different types of tumors was accessed through the CellMiner interface (https://discover.nci.nih.gov/cellminer)^25^. The Pearson correlation analysis was run to explore the relationship between the expression of 9 prognostic genes and 263 drugs approved by the FDA or clinical trials.

### Assessment of the prognostic Nomogram Performance

The risk score and clinical characteristics were used to construct a multivariate Cox regression model. Then the R package "regplot" was used to create a Nomogram, a clinically accurate quantitative tool for predicting the survival of OSCC patients at 1, 2, 3 years. To verified the accuracy and optimality of Nomogram in predicting survival, we drew the calibration curves and the ROC curves for OS at 1, 2, and 3 years. Finally, we compared the ROC curves of Nomogram, risk score, and other clinical characteristics.

### Statistical analysis

Depending on the features of the distribution, differences between continuous variables were classified by the Student's t-test or Mann–Whitney test and differences between categorical variables were classified by chi-square test or Fisher's exact test determined. To build a risk model based on the PR-DE-FRGs, we successively used univariate Cox regression analysis and lasso regression. The COX multivariate regression method was used to construct the Nomogram. The OS between groups was distinguished by Kaplan–Meier curve of log-rank test. The predictive power of different factors on the prognosis was judged by ROC curve. Risk score for an independent predictor of survival plays a useful role, we used univariate and multivariate Cox regression analysis to determine. The correlation between the variables was confirmed by Spearman or Pearson correlation analysis. It is stated that all analysis techniques were provided by the R programming language (version 4.0.3, www.r-project.org/) and SPSS Statistics (version 22, www.ibm.com/products/spss-statistics).

## Results

### Identification of DE-FRGs

We extracted the gene expression quantification of 246 FRGs from the TCGA cohort to identify 165 DE-FRGs, in which 123 were up-regulated genes and 42 were down-regulated genes (Supplementary Fig. S2A). Similarly, we obtained the gene expression quantification of 234 FRGs from the GSE30784 cohort to identify 155 DE-FRGs, in which 102 were up-regulated genes and 53 were down-regulated genes (Supplementary Fig. S2B).

### Establishment of the regulatory network and prognostic model

**Figure 2:**
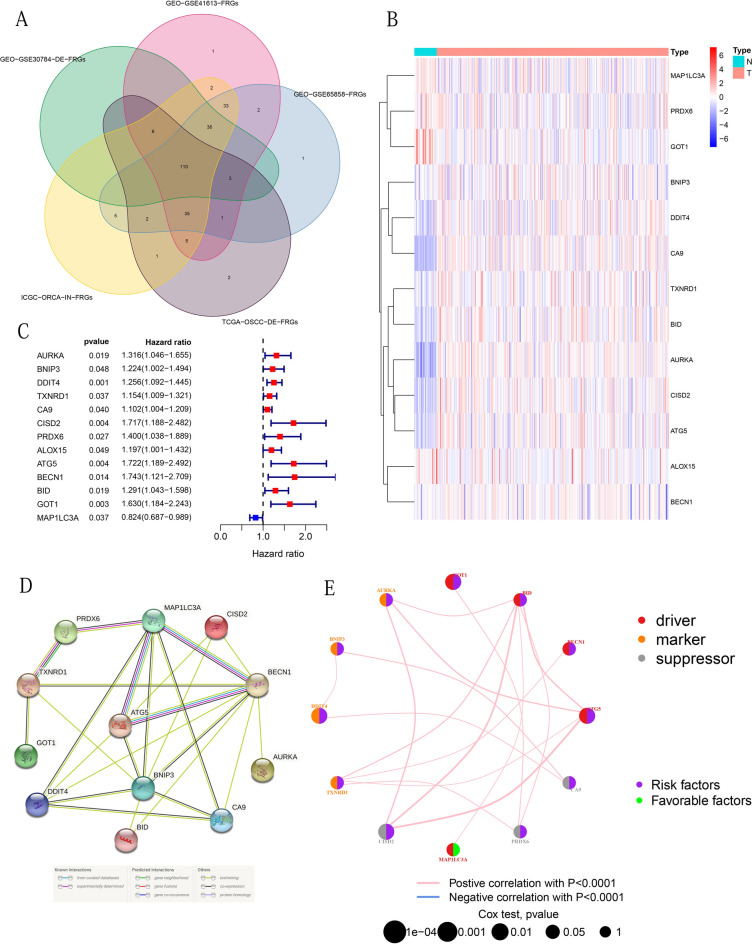
The identification of PR-DE-FRGs that build the associated network. (**A**) Overlap the DE-FRGs obtained from the TCGA and GSE30784 cohorts and FRGs obtained from the GSE41613, GSE65858, and ICGC-ORCA-IN cohorts, thereby obtained overlapping DE-FRGs. (**B**) Heat map of 13 PR-DE-FRGs expression profiles. (**C**) Forest plot of the results of OS univariate COX analysis on 13 PR-DE-FRGs. (**D**) PPI was based on the 12 PR-DE-FRGs (Except for ALOX15) used in building the model. The different colored lines between the nodes at the bottom of the graph indicate different sources of evidence. (**E**) Correlation network of 12 PR-DE-FRGs (Except for ALOX15). The left part of the circle in the figure implies the attributes of genes, and the right part implies the influence of genes on prognosis. Driver refers to the gene that drives ferroptosis; Suppressor refers to the gene that suppresses ferroptosis; and Marker refers to the gene that prompts ferroptosis to occur. The above figures were drawn using R programming language (version 4.0.3, www.r-project.org/).

**Figure 3:**
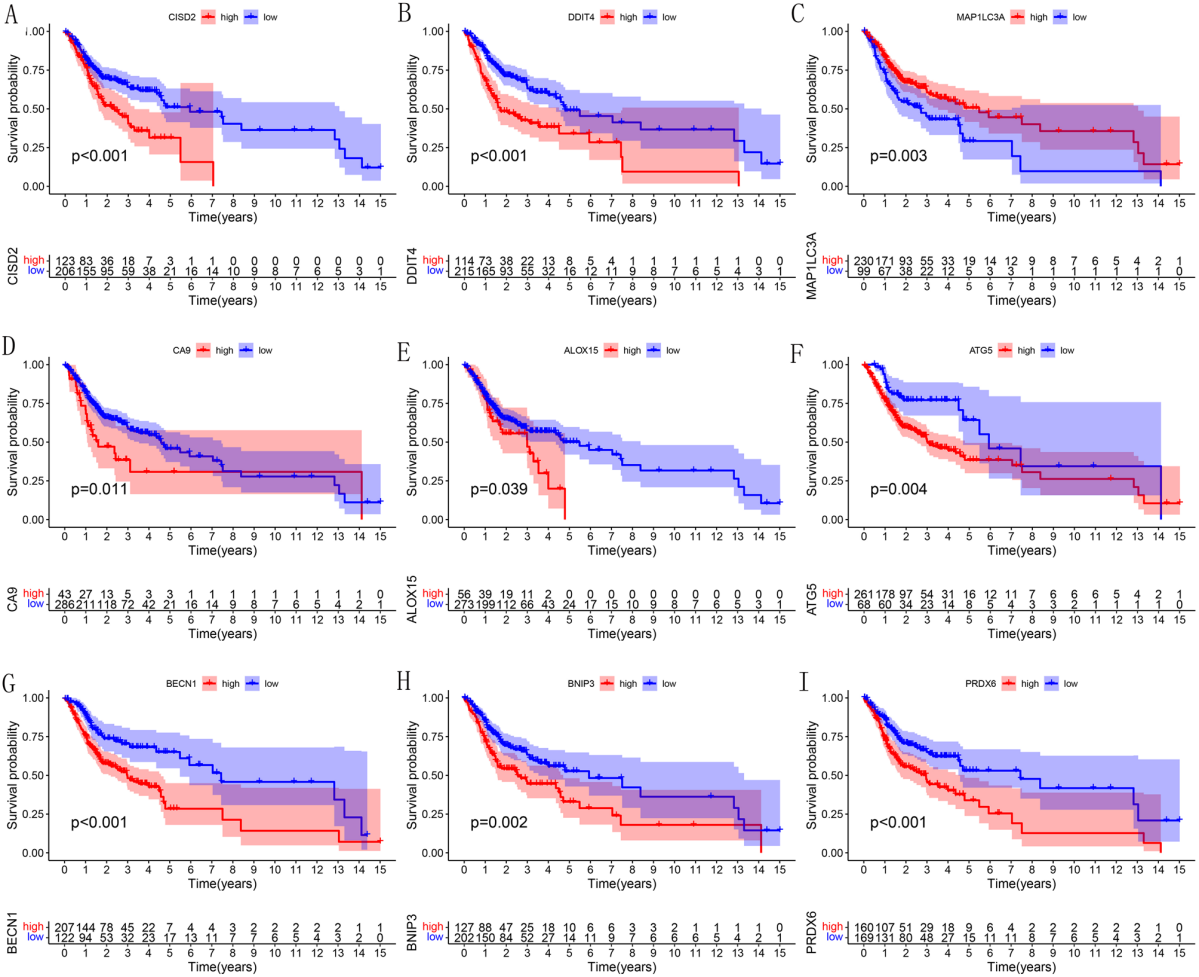
Survival curves of 9 PR-DE-FRGs for constructing the model. (**A**) CISD2. (**B**) DDIT4. (**C**) MAP1LC3A. (**D**) CA9. (**E**) ALOX15. (**F**) ATG5. (**G**) BECN1. (**H**) BNIP3. (**I**) PRDX6. The above figures were drawn using R programming language (version 4.0.3, www.r-project.org/).

We overlapped the DE-FRGs obtained from the TCGA and GSE30784 cohorts and FRGs obtained from the GSE41613, GSE65858, and ICGC-ORCA-IN cohorts, and finally obtained 110 overlapping DE-FRGs (Fig. 2A). By integrating RNA expression and clinical data from OSCC patients in the four cohorts, we obtained 329 samples from the TCGA cohort, 97 samples from the GSE41613 cohort, 83 samples from the GSE65858 cohort and 28 samples from the ICGC-ORCA-IN cohort. The clinical characteristics were shown in Tables 1–2. We performed univariate cox analysis of OS on 110 crossed DE-FRGs and found 13 PR-DE-FRGs (Fig. 2B,C). Except for MAP1LC3A (HR < 1), the remaining 12 genes (AURKA, BNIP3, DDIT4, TXNRD1, CA9, CISD2, PRDX6, ALOX15, ATG5, BECN1, BID and GOT1, HR > 1) were identified as prognostic risk factors (Fig. 2C). We used the training set to perform lasso regression analysis based on the optimal value of λ (Supplementary Fig. S1A,B) to determine 9 highly correlated PR-DE-FRGs used to construct the prognostic model. The risk score was calculated by the following formula: risk score = (0.11544*BNIP3 expression value) + (0.12777* DDIT4) + (0.05508*CA9) + (0.32123*CISD2) + (0.20484* PRDX6) + (0.10414* ALOX15) + (0.07954*ATG5) + (0.43047*BECN1) + (−0.09567*MAP1LC3A). The corresponding coefficients of PR-DE-FRGs used to construct the model were shown in Supplementary Table 1. Six hub genes (ATG5, BNIP3, MAP1LC3A, BECN1, CA9, and DDIT4) were selected by establishing the PPI for 12 PR-DE-FRGs (Fig. 2D). The results of 12 PR-DE-FRGs with correlation were shown on the correlation network diagram (Fig. 2E). In addition, the Kaplan–Meier survival curves of 9 PR-DE-FRGs demonstrated that better OS with low expression of CISD2, DDIT4, CA9, ALOX15, ATG5, BECN1, BNIP3 and PRDX5 expect for MAP1LC3A (p < 0.05, Fig. 3).

**Table 1:** Clinical characteristics of the OSCC samples in training, test, whole set from TCGA.

	Training set (n = 200)	Test set (n = 129)	Whole set (n = 329)
**Gender **(**%**)			
Male	139 (69.5%)	87 (67.4%)	226 (68.7%)
Female	61 (30.5%)	42 (32.6%)	103 (31.3%)
**Age **(**median**,** range**)	61 (24–88)	62 (19–90)	61 (19–90)
**Survival status**			
OS-days (median, range)	582 (11–5480)	560 (1–5252)	577 (1–5480)
OS-state(alive(%)/dead(%))	111 (55.5%)/89 (44.5%)	81 (62.8%)/48 (37.2%)	192 (58.4%)/137 (41.6%)
**Grade**(**%**)			
1	31 (15.5%)	20 (15.5%)	51 (15.5%)
2	122 (61.0%)	79 (61.2%)	201 (61.1%)
3	43 (21.5%)	24 (18.6%)	67 (20.4%)
4	1 (0.5%)	1 (0.8%)	2 (0.6%)
Unknown	3 (1.5%)	5 (3.9%)	8 (2.4%)
**Stage**(**%**)			
I	10 (5.0%)	9 (7.0%)	19 (5.8%)
II	32 (16.0%)	21 (16.3%)	53 (16.1%)
III	42 (21.0%)	18 (14.0%)	60 (18.2%)
IV	96 (48.0%)	71 (55.0%)	167 (50.8%)
Unknown	20 (10.0%)	10 (7.7%)	30 (9.1%)
**T**(**%**)			
1	17 (8.5%)	13 (10.1%)	30 (9.1%)
2	63 (31.5%)	35 (27.1%)	98 (29.8%)
3	40 (20.0%)	26 (20.1%)	66 (20.1%)
4	64 (32.0%)	46 (35.7%)	110 (33.4%)
Unknown	16 (8.0%)	9 (7.0%)	25 (7.6%)
**M**(**%**)			
0	74 (37.0%)	46 (35.7%)	120 (36.5%)
1	0 (0.0%)	0 (0.0%)	0 (0.0%)
Unknown	126 (63.0%)	83 (64.3%)	209 (63.5%)
**N**(**%**)			
0	72 (36.0%)	47 (36.4%)	119 (36.2%)
1	35 (17.5%)	14 (10.9%)	49 (14.9%)
2	63 (31.5%)	43 (33.3%)	106 (32.2%)
3	1 (0.5%)	1 (0.8%)	2 (0.6%)
Unknown	29 (14.5%)	24 (18.6%)	53 (16.1%)

**Table 2:** Clinical characteristics of the OSCC samples in 3 sets from GEO and ICGC set.

	GEO		ICGC	
	GSE41613 set (n = 97)	GSE65858 set (n = 83)	GSE30784 set (n = 167)	ORCA-IN set(n = 28)
**Gender **(**%**)				
Male	66 (68.0%)	64 (77.1%)	120 (71.9%)	22 (78.6%)
Female	31 (32.0%)	19 (22.9%)	47 (28.1%)	6 (21.4%)
**Age **(**%**)				
–59	50 (51.5%)	47 (56.6%)	90 (53.9%)	22 (78.6%)
60-	47 (48.5%)	36 (43.4%)	77 (46.1%)	6 (21.4%)
**Survival status**				
OS-months (median, range)	54.41 (0.46–85.03)	27.32 (1.51–67.69)	–	12.62 (3.81–27.16)
OS-state(alive(%)/dead(%))	46 (47.4%)/51 (52.6%)	56 (67.5%)/27 (32.5%)	–	20 (71.4%)/8 (28.6%)

### Prognostic model performed well in the assessment of predictive power

**Figure 4:**
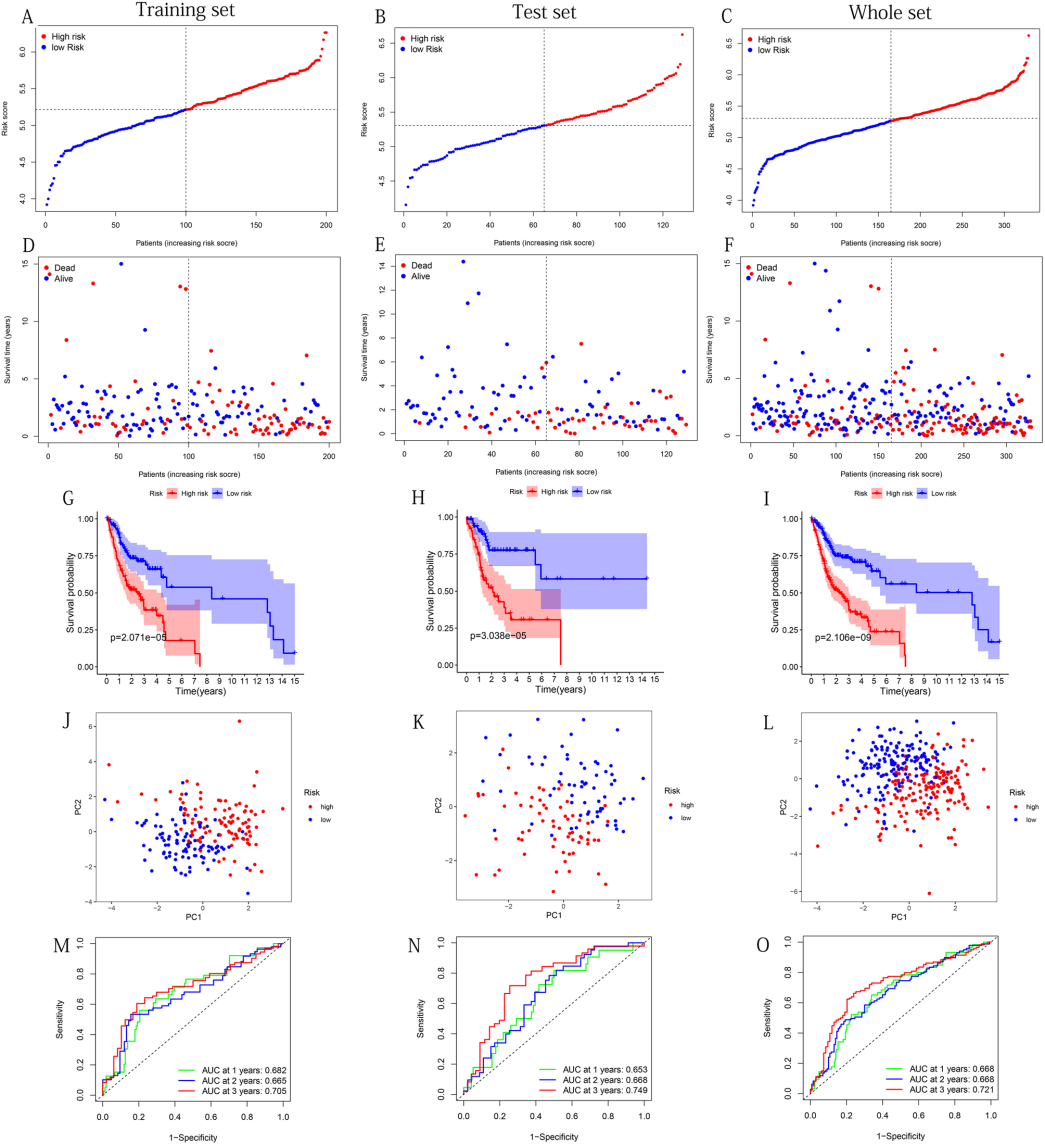
Use the training, test and the whole sets from TCGA to construct the risk plots, survival point maps, survival curves, PCA discrete trend charts, and ROC curves. (**A**–**C**) Risk plots. (**D**–**F**) Survival point maps. (**G**–**I**) Survival curves. (**J**–**L**) PCA discrete trend charts. (**M–O**) ROC curves at 1, 2, and 3 years. The above figures were drawn using R programming language (version 4.0.3, www.r-project.org/).

**Figure 5:**
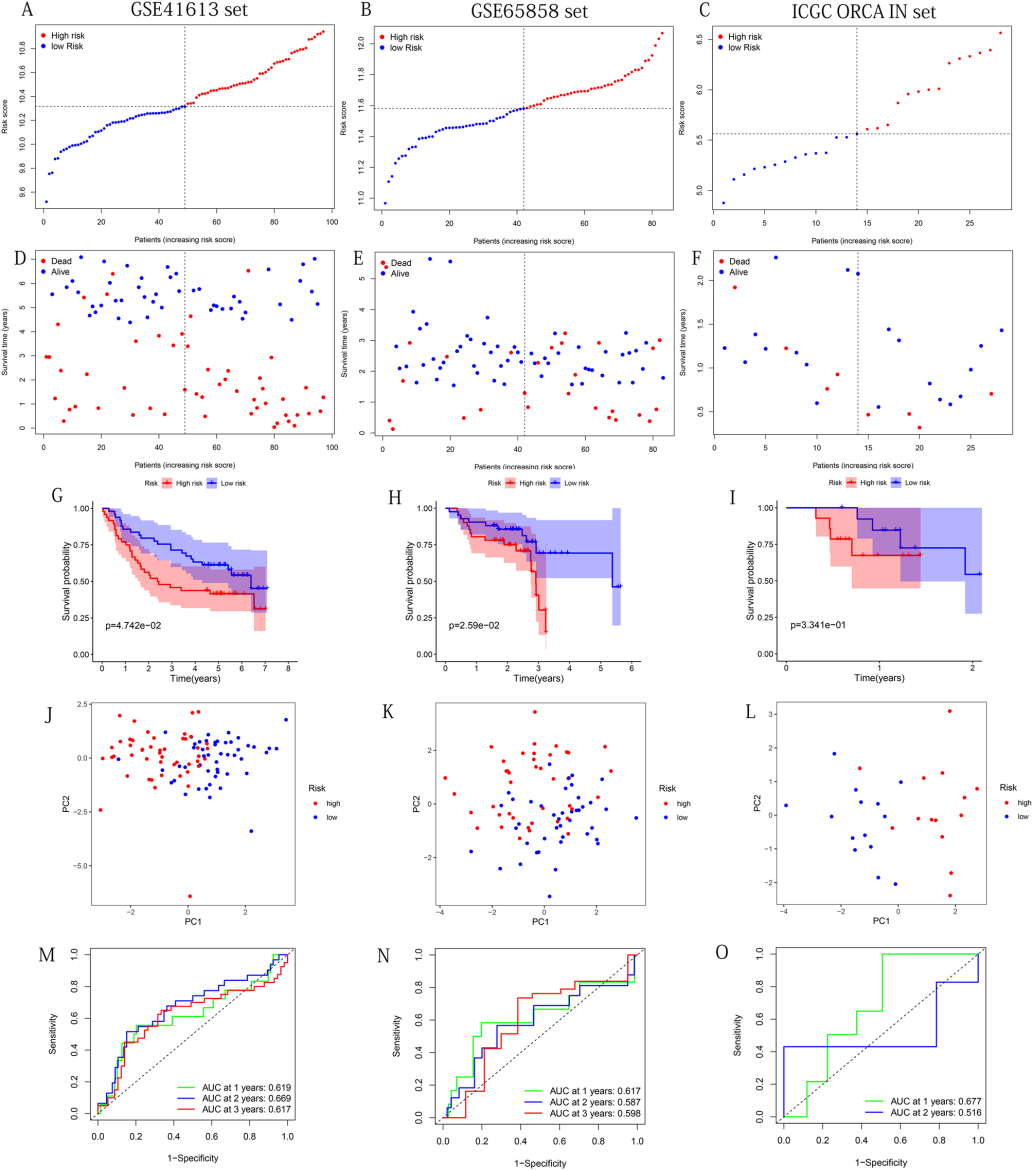
Use the GSE41613, GSE65858 and ICGC-ORCA-IN sets to construct the risk plots, survival point maps, survival curves, PCA discrete trend charts, and ROC curves. (**A**–**C**) Risk plots. (**D**–**F**) Survival point maps. (**G**–**I**) Survival curves. (**J**–**L**) PCA discrete trend charts. (**M**–**O**) ROC curves at 1, 2, and 3- years. The above figures were drawn using R programming language (version 4.0.3, www.r-project.org/).

In the TCGA's three datasets and GSE41613 and GSE65858 cohorts, high-risk groups had more deaths than low-risk groups (Figs. 4A–F,5A–F). The results of Kaplan–Meier test showed that except for the results were not significantly different in ICGC-ORCA-IN cohorts, patients in the high-risk group had a significantly lower survival probability in the other five groups (Figs. 4G–I; 5G–I). The PCA demonstrated that the cases from the high-risk and the low-risk group in the 6 groups were distributed in discrete directions (Figs. 4J–L, 5J–L). Univariate Cox regression analysis showed that the training, test, and whole sets' risk score were significantly correlated with OS (Table 3). After adjusting for other clinical confounding factors, the risk score was still determined as an independent prognostic factor of OS in each group, with multivariate Cox regression analysis (Table 3). To verify the optimality of the model, we drew the ROC curves based on the training, test, and whole sets. AUC (at 1, 2, and 3 years) values were above 0.65 (Fig. 4M–O), indicating that the model has satisfactory predictive power. The ROC curves of the three external validation cohorts still showed that the model had good predictive capability (Fig. 5M–O). It was worth mentioning that consistent results were obtained in the three sets from TCGA: The AUC values of risk score were the largest among all factors, which indicated that the model had the superb prognosis prediction effect (training set: Fig. 15A–C; test set: Fig. 15D–F; Whole set: Fig. 15G–I).

**Table 3:** The results of univariate and multivariate Cox regression analysis including clinical factors and risk scores in three set from TCGA.

	Univariate cox		P^1^	Multivariate cox		P^2^
	HR	95%CI		HR	95%CI	
**Training set**						
Age	1.07	0.70–1.63	0.751	1.23	0.701–2.141	0.475
Gender	0.93	0.60–1.44	0.734	1.00	0.575–1.736	0.998
Grade	1.55	1.14–2.12	**0.005**	1.63	1.045–2.552	**0.031**
Stage	1.39	1.07–1.81	**0.013**	0.96	0.521–1.786	0.908
T	1.40	1.12–1.75	**0.003**	1.44	0.938–2.216	0.095
N	1.38	1.06–1.80	**0.017**	1.25	0.871–1.808	0.224
Riskscore	3.70	2.14–6.41	** < 0.001**	2.79	1.432–5.446	**0.003**
**Test set**						
Age	1.81	0.99–3.31	0.053	1.81	0.839–3.93	0.137
Gender	0.87	0.48–1.56	0.639	1.37	0.648–2.91	0.408
Grade	0.84	0.53–1.33	0.455	0.76	0.412–1.41	0.391
Stage	1.69	1.16–2.45	**0.006**	1.20	0.558–2.57	0.644
T	1.43	1.05–1.93	**0.022**	1.07	0.694–1.66	0.754
N	1.84	1.28–2.64	** < 0.001**	1.49	0.937–2.38	0.092
Riskscore	4.62	2.32–9.22	** < 0.001**	3.88	1.60–9.42	**0.003**
**Whole set**						
Age	1.31	0.93–1.84	0.120	1.35	0.88–2.06	0.167
Grade	0.89	0.63–1.26	0.516	1.16	0.75–1.80	0.509
Gender	1.23	0.95–1.59	0.116	1.18	0.83–1.66	0.358
Stage	1.52	1.22–1.88	** < 0.001**	1.09	0.69–1.74	0.706
T	1.41	1.18–1.69	** < 0.001**	1.24	0.92–1.69	0.163
N	1.53	1.24–1.89	** < 0.001**	1.35	1.03–1.77	**0.029**
Riskscore	3.77	2.47–5.76	** < 0.001**	3.19	1.93–5.27	** < 0.001**

Significant values are in bold.

### Immunohistochemistry images verified the up-regulation of 4 PR-DE-FRGs in OSCC tissues

**Figure 6:**
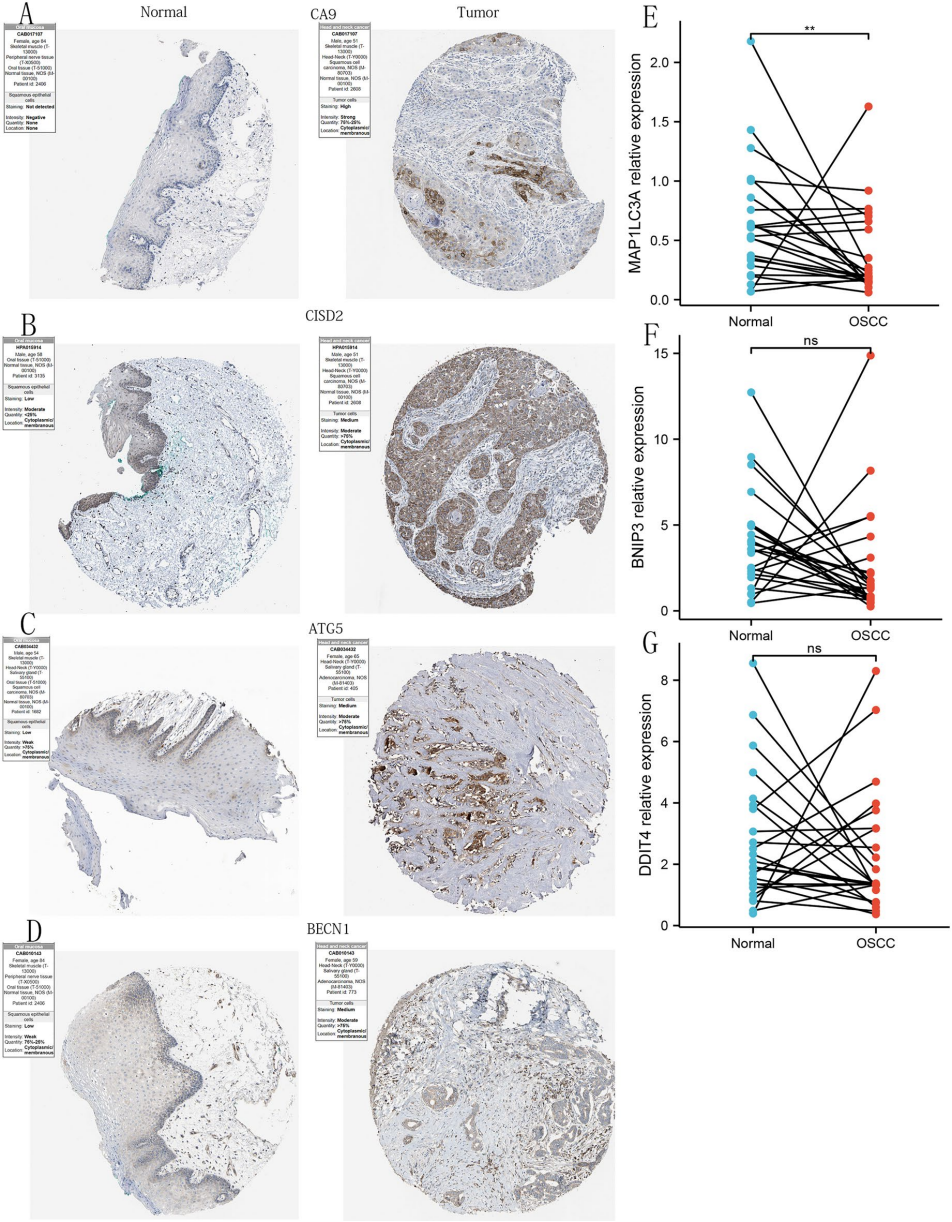
IHC images based on the expression of 4 PR-DE-FRGs proteins and the relative mRNA expression of MAP1LC3A, BNIP3 and DDIT4 detected by qRT-PCR in OSCC and normal oral tissues. (**A**) CA9. (**B**) CISD2. (**C**) ATG5. (**D**) BECN1. (**E**) MAP1LC3A. (**F**) BNIP3. (**G**) DDIT4. The above figures were drawn using R programming language (version 4.0.3, www.r-project.org/).

After comparing the immunohistochemical staining images of 7 DE-FRGs (CA9, CISD2, ATG5, BECN1, BNIP3, DDIT4 and MAP1LC3A) protein expression in cancerous and normal oral tissues (Fig. 6), we found that CA9, CISD2, ATG5 and BECN1 protein expression is higher in the OSCC organization (Fig. 6A–D). These indicated the correctness of our analysis of the differential expression of these genes. And BNIP3, DDIT4 and MAP1LC3A had no difference in protein expression (Supplementary Fig. S3A–C).

### QRT-PCR for verified the differential expression of BNIP3, DDIT4 and MAP1LC3A

From paired differential comparisons, we found that the relative mRNA expression of MAP1LC3A in OSCC was lower than in normal oral tissues, which was consistent with the shared data analysis results (Fig. 6E). Unfortunately, we have not found the difference in the relative mRNA expression of BNIP3 and DDIT4 between OSCC and oral normal tissues (Fig. 6F–G).

### Prognostic model associated with clinicopathological features

**Figure 7:**
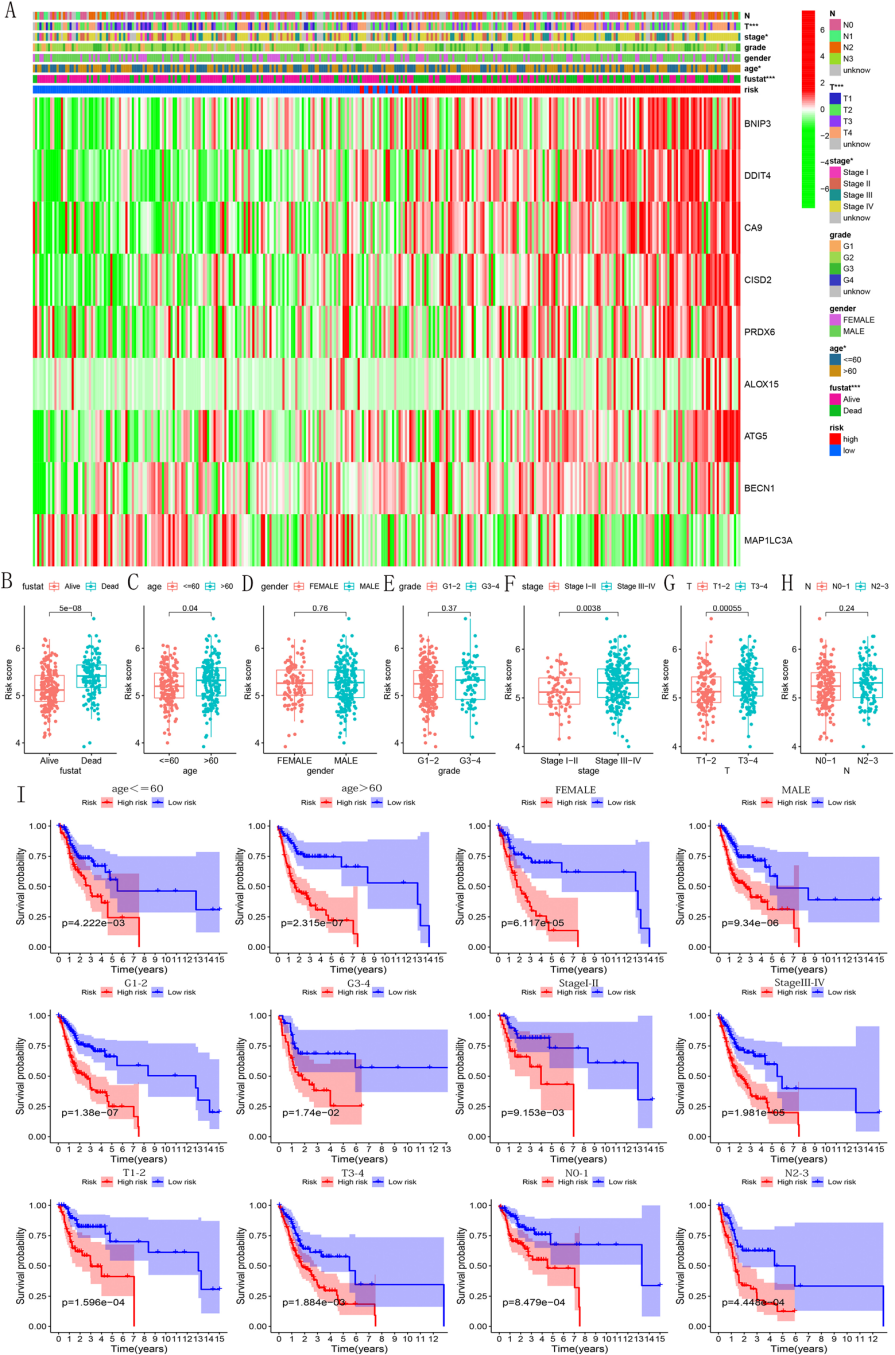
Comparison of risk scores between different subgroups of each clinical feature. (**A**) When increasing the risk score, the layout of the different subtypes of each clinical feature is displayed. (**B**–**H**) Comparison of risk scores between the different subtypes of each clinicopathological feature. (**I**) Analysis of the survival probability for each subgroup with different clinical features.

The heat map roughly showed the distribution of clinical features of each sample (Fig. 7A). Next, after analyzing the relationship between risk score and various clinical characteristics, we found that survival status (Fig. 7B, p < 0.05), age (Fig. 7C, p < 0.001), stage (Fig. 7F, p < 0.01) and T staging (Fig. 7G, p < 0.001) were highly correlated with risk scores. In addition, the risk score of samples in the age > 60-year-old group, death group, stage III-IV group, and T3–4 group were higher. Higher expression of CISD2/MAP1LC3A/PRDX6 was observed at higher grade (Supplementary Fig. S4C,G,I). CISD2/ATG5/DDIT4 was more expressed in stage III-IV population (Supplementary Fig. S4B,D,F). None of the other results showed statistical significance (Supplementary Figs. S4A,E,H and S5A–I). These results supported the potential role of CISD2/MAP1LC3A/PRDX6/ATG5/DDIT4 in the proliferation and invasion of OSCC cells. By analyzing the survival probability of samples in each subgroup with different clinical characteristics, it was found that the prognostic model still maintains the ability to predict the prognosis. It is worth mentioning that in each subgroup, the OS of patients in the low-risk group was higher than that of the high-risk group (Fig. 7I, p < 0.05).

### Gene set enrichment analysis results

**Figure 8:**
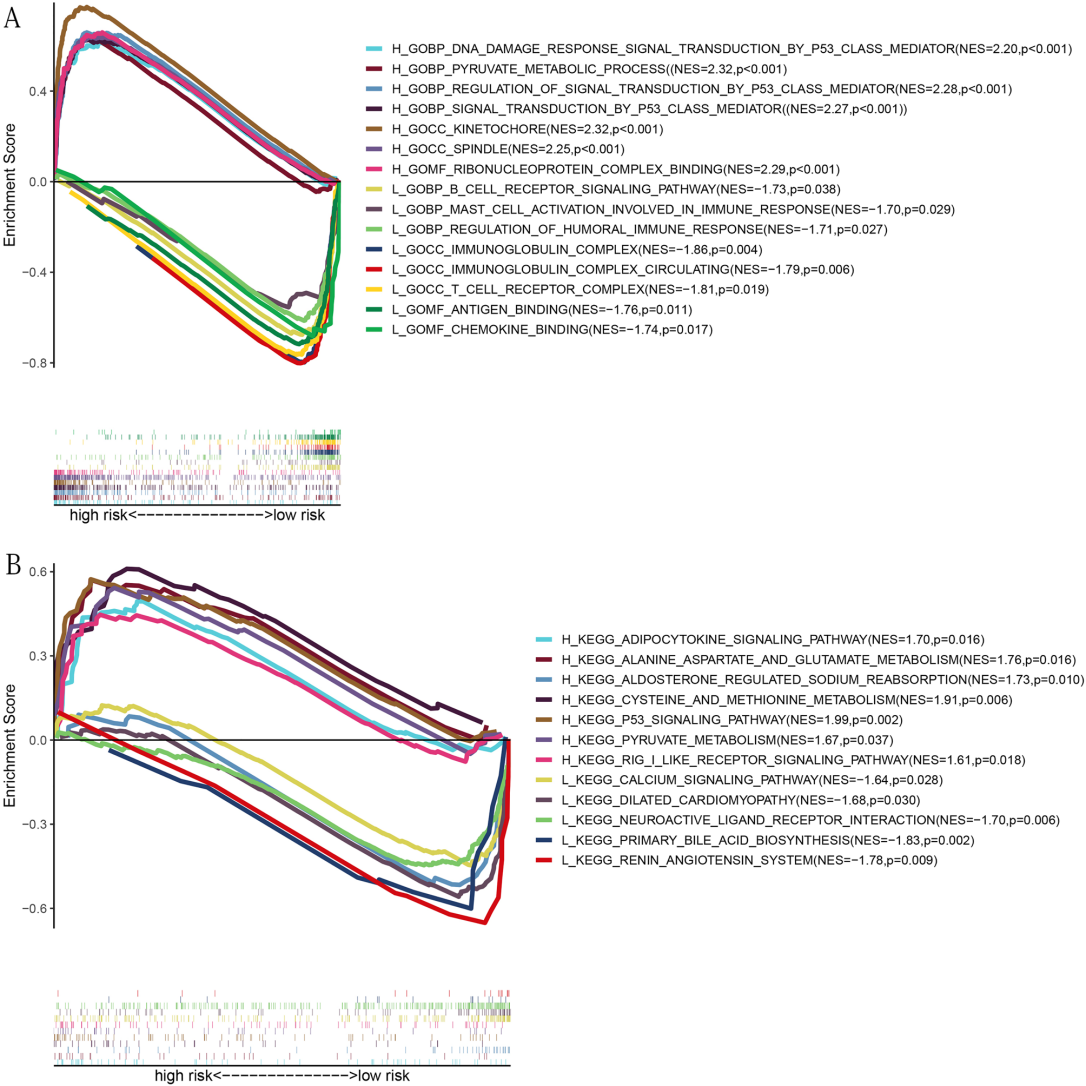
GSEA based on 9 PR-DE-FRGs. (**A**) GO enrichment analysis results in high-risk and low-risk groups. (**B**) KEGG enrichment analysis results in high-risk and low-risk groups.NES: normalized enrichment score; H and L represents high-risk group and low-risk group, respectively. NES and p-value were marked behind each pathway or function. The above figures were drawn using R programming language (version 4.0.3, www.r-project.org/).

The results of GO and KEGG enrichment were shown in Fig. 8A,B, respectively. Interestingly, the high-risk group was enriched in many P53-related biological processes (including signal transduction by p53 class mediator and regulation of signal transduction by p53 class mediator) and ferroptosis-related biological processes (pyruvate metabolic process), while the low-risk group was enriched in a large number of immune-related cell components (including immunoglobulin complex, immunoglobulin complex, circulating, and T cell receptor complex), molecular function (including antigen binding, and chemokine binding), and biological process (including B cell receptor signaling pathway, mast cell activation involved in immune response, and regulation of humoral immune response) (Fig. 8A). The result of further KEGG enrichment analysis was shown in Fig. 8B. Expectedly, many ferroptosis-related and P53-related pathways were enriched (including Adipocytokine signaling pathway, Alanine, aspartate, and glutamate metabolism, Cysteine and methionine metabolism, p53 signaling pathway, Pyruvate metabolism, Calcium signaling pathway) (Fig. 8B).

### Prognostic model associated with immune microenvironment

**Figure 9:**
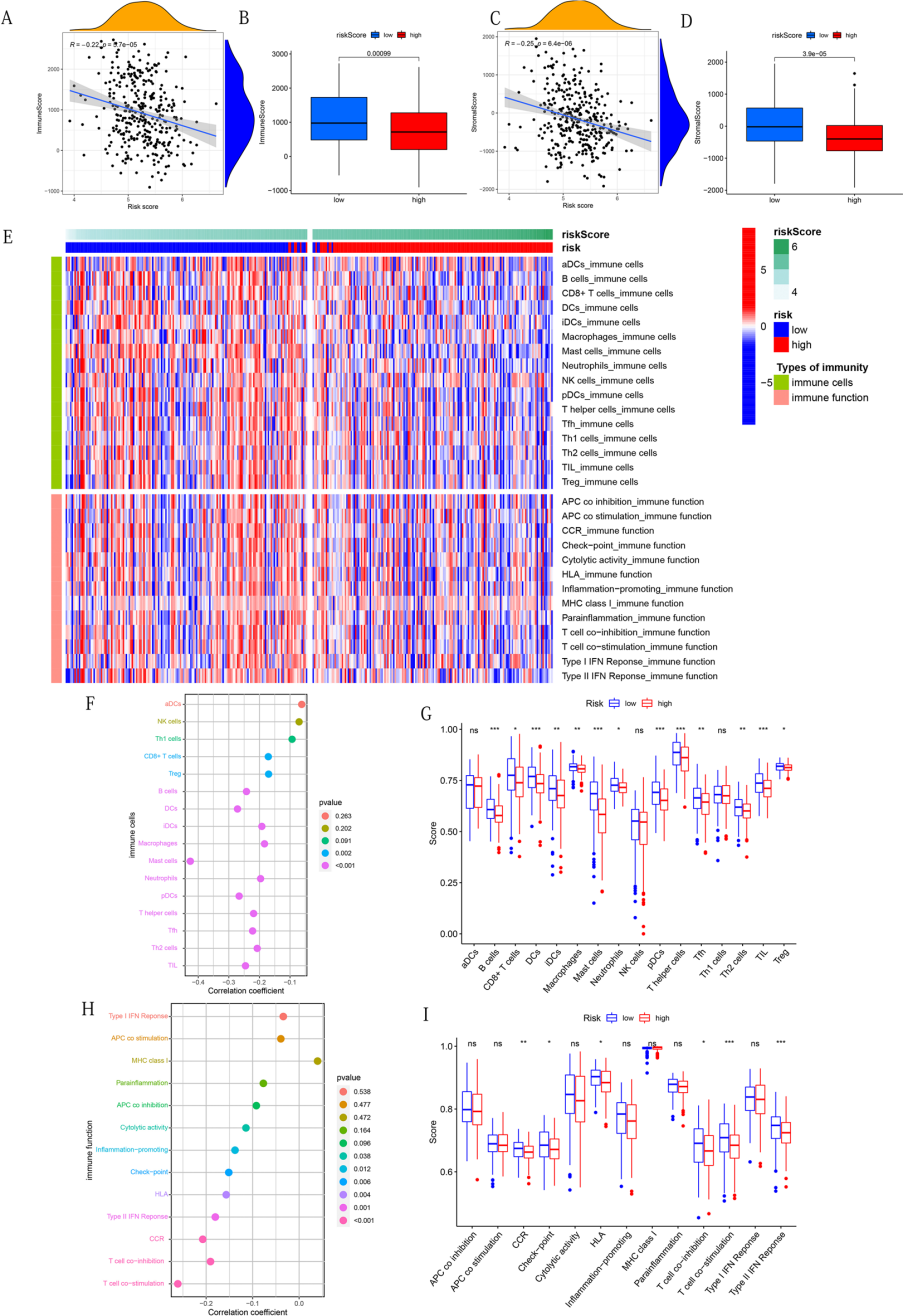
Analysis of the correlation between risk score and immune score, stromal score, 16 immune infiltrating cells and 13 immune functions, and further comparison of them between the high-risk group and the low-risk group. (**A**,**B**) Immune score. (**C**,**D**) Stromal score. (**E**) Heat map of the distribution difference of 16 kinds of immune cells and 13 kinds of immune functions. (**F**,**G**) 16 immune infiltrating cells. (**H**,**I**) 13 immune functions. The symbol above the histogram indicates the significance of the difference: *p < 0.05; **p < 0.01; ***p < 0.001; ns: no significance. The above figures were drawn using R programming language (version 4.0.3, www.r-project.org/).

Since GO enrichment analysis found that the low-risk group was enriched with numerous immune-related biological functions and pathways, we conducted an in-depth analysis to further understand whether the risk score is related to the immune microenvironment. First, the correlation between overall immune cells or stromal cells and risk scores were analyzed. The results showed that the risk score was significantly negatively correlated with the immune score or stromal score (P < 0.001, Fig. 9A,C). The relevant results were supported by further difference analysis (Fig. 9B,D). The heatmap in Fig. 9E showed an overview of the 16 immune cell scores and 13 immune function scores for each sample with a different risk score. Next, we deeply analyzed the correlation between 16 types of immune cells or 13 types of immune-related functions and risk score. CD8+ T cells, regulatory T cells (Treg), B cells, dendritic cells (DCs), immature dendritic cells (iDCs), Macrophages, Mast cells, Neutrophils, plasmacytoid dendritic cells (pDCs), T helper cells, T-helper follicular (Tfh) cells, type 2 helper T (Th2) cells, and tumor-infiltrating lymphocytes (TIL) scores were negatively correlated with risk scores (P < 0.05, Fig. 9F). The scores of immune cells in the above 13 were significantly higher in the low-risk group (P < 0.05, Fig. 9G). Cytolytic activity, Inflammation − promoting, Check − point, human leukocyte antigen (HLA), Type II IFN Response, Chemokine receptors (CCR), T cell co − stimulation, and T cell co − inhibition were observed to be negatively correlated with risk score (P < 0.05, Fig. 9H). In addition, it was observed that CCR, Check-point, HLA, T cell co-stimulation, T cell co-inhibition, and Type II IFN Response scores were higher in the low-risk group (P < 0.05, Fig. 9I).

### Prognostic model associated with mutational signatures

**Figure 10:**
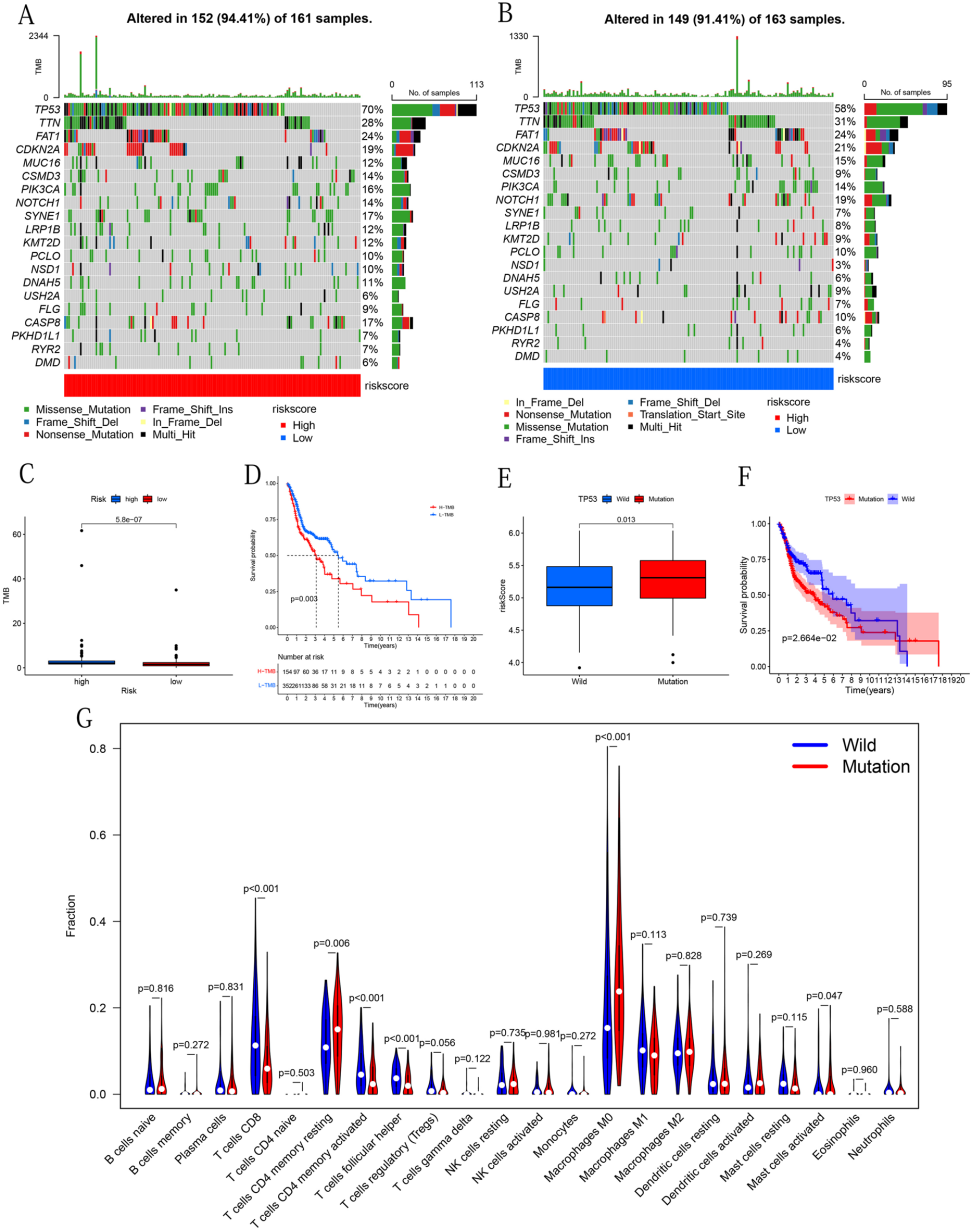
Mutation analysis. (**A**,**B**) The waterfall plots describe the mutation layout of samples in the high-risk and the low-risk groups, respectively. The right side of the waterfall plots are the mutation frequency, which is the basis of gene sequencing. At the bottom of the figures are the type of mutation. The histograms above show the TMB statistics for each sample. (**C**) TMB comparison between high-risk and low-risk groups. (**D**) Survival difference curve based on samples of high TMB and low TMB groups. (**E**) Risk score comparison between TP53 mutation and TP53 wild groups. (**F**) Survival difference curve based on samples of TP53 mutation and TP53 wild groups. (**G**) Comparison of 22 immune cells between TP53 mutation and TP53 wild groups. The above figures were drawn using R programming language (version 4.0.3, www.r-project.org/).

We drew waterfall plots and boxplots to explore differences in mutation characteristics between the high-risk and low-risk group. The waterfall chart displayed the mutations of the top 20 most common genes in 152 samples in the high-risk group and 149 in the low-risk group (Fig. 10A,B). The 10 most frequently mutated genes from the TCGA cohort were TP53 (66%), TTN (35%), FAT1 (21%), CDKN2A (20%), MUC16 (17%), CSMD3 (16%), PIK3CA (16%), NOTCH1 (16%) and SYNE1 (15%). The results showed that the most frequently mutated gene in the two groups was the TP53 gene. The boxplot showed that the high-risk group had a higher TMB than the low-risk group (Fig. 10C). Surprisingly, the subsequent survival analysis showed that the high TMB sample had a lower survival probability (Fig. 10D). We also analyzed the relationship between the mutant gene and the risk score. We then found that the TP53 mutation group had a higher risk score (Fig. 10E). The subsequent survival analysis showed that the TP53 mutation group had worse OS (Fig. 10F). Finally, we also analyzed the difference of immune cells between the two groups. The lollipop chart showed that in the wild group T cells CD8, T cells CD4 memory activated, T cells follicular helper, and mast cells activated ratio were higher, while the ratio of T cells CD4 memory resting and macrophage M0 were higher in the mutant group (Fig. 10G).

### Prognostic model associated with stem-cell characteristics

**Figure 11:**
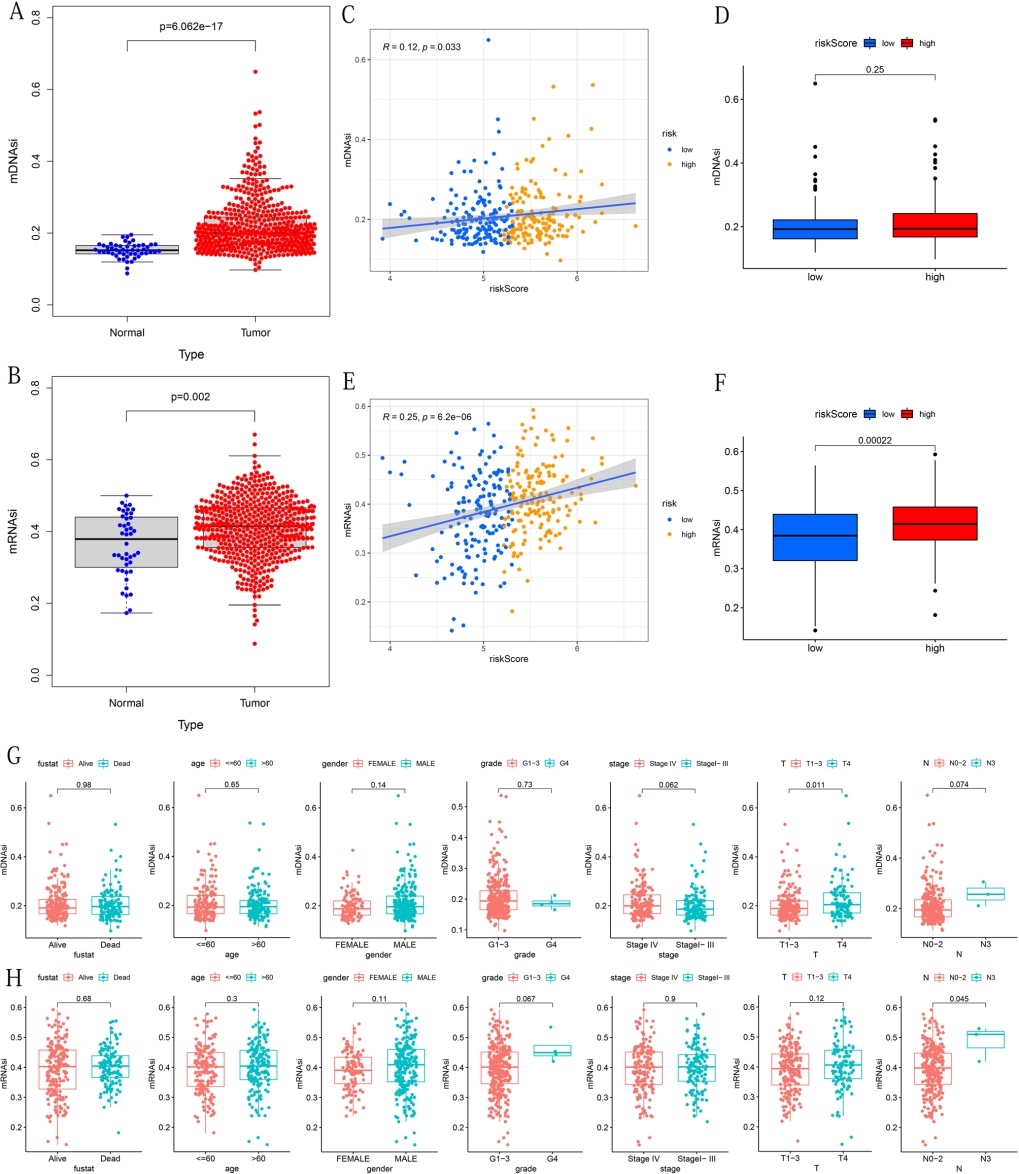
Correlation between risk score, clinicopathological characteristics and mDNAsi/mRNAsi. (**A**,**B**) Comparison of mDNAsi/mRNAsi between cancerous and normal groups. (**C**,**E**) Correlation between mDNAsi/mRNAsi and risks core. (**D**,**F**) Comparison of mDNAsi/mRNAsi between high- and low-risk groups. (**G**,**H**) Comparison of mDNAsi/mRNAsi between different subtypes of each clinicopathological feature. The above figures were drawn using R programming language (version 4.0.3, www.r-project.org/).

Cell stemness is closely related to the proliferation and invasion of tumor cells. The previous stratified analyses showed a strong relationship between risk score and stage/T stage (Fig. 7F–G). Therefore, we further analyzed stemness characteristics. Firstly, we analyzed the mDNAsi/mRNAsi difference between the cancerous and the normal groups and found that the mDNAsi and mRNAsi of the tumor group were significantly higher (Fig. 11A,B). Then, we studied the correlation between mDNAsi/mRNAsi and risk score (Fig. 11C,E) and the difference of mDNAsi/mRNAsi between different risk groups (Fig. 11D,F). The results showed a positive correlation between mDNAsi/mRNAsi and risk score, and the mRNAsi of patients in the high-risk group was significantly higher. Finally, by analyzing the differences in mDNAsi/mRNAsi between the different subtypes of each clinicopathological feature, we found that mDNAsi is higher in patients with stage T4 (Fig. 11G), and patients with stage N3 had significantly higher mRNAsi (Fig. 11H).

### Risk score closely correlated with ICIs or M6A related gene expression

**Figure 12:**
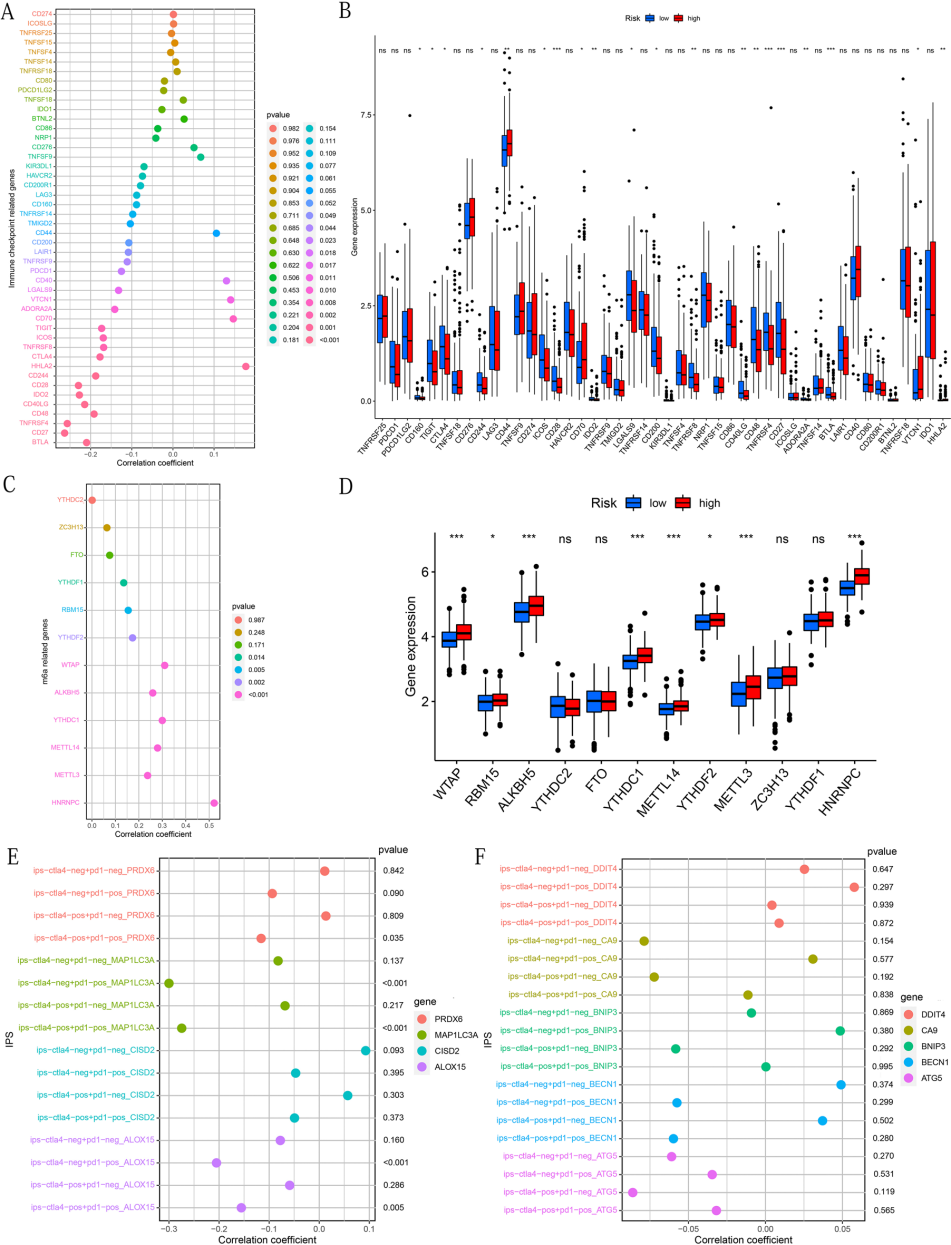
Prognosis model associated with ICIs-related genes, m6a-related genes and IPS. The correlation between risk score and ICIs-related genes, m6a-related genes and their differences in high and low risk groups: (**A**,**B**) The expression value of ICIs-related genes. (**C**,**D**) The expression value of m6a-related genes. The relationship between IPS and 9 PR-DE-FRG expressions: (**E**) PRDX6, MAP1LC3A, CISD2 and ALOX15. (**F**) DDIT4, CA9, BNIP3, BECN1 and ATG5. The above figures were drawn using R programming language (version 4.0.3, www.r-project.org/).

Given the differences in check-point found in different risk groups (Fig. 9I) and the increasing importance of ICIs in immunotherapy, we analyzed the correlation between 46 ICIs-related genes expression values and risk scores and their comparison in different risk groups (Fig. 12A-B). It can be understood from Fig. 12A that the expression of LAIR1, TNFRSF9, PDCD1, LGALS9, ADORA2A, TIGIT, ICOS, TNFRSF8, CTLA4, CD244, CD28, IDO2, CD40LG, CD48, TNFRSF4, CD27, BTLA were negatively correlated with the risk score. The expression levels of CD40, VTCN1, CD70, and HHLA2 were positively correlated with the risk score (Fig. 12A). Except for IR1, TNFRSF9, PDCD1, and CD40, the correlation results were all supported by further difference analysis (Fig. 12B). Besides that, we also found that the expression levels of YTHDF1, RBM15, YTHDF2, WTAP, ALKBH5, YTHDC1, METL14, METL3, and HNRNPC were all positively correlated with the risk score (Fig. 12C). Except for YTHDF1, the correlation results of other m6a-related genes were verified in the difference analysis (Fig. 12D).

### The prognostic model demonstrated excellent clinical guidance value

**Figure 13:**
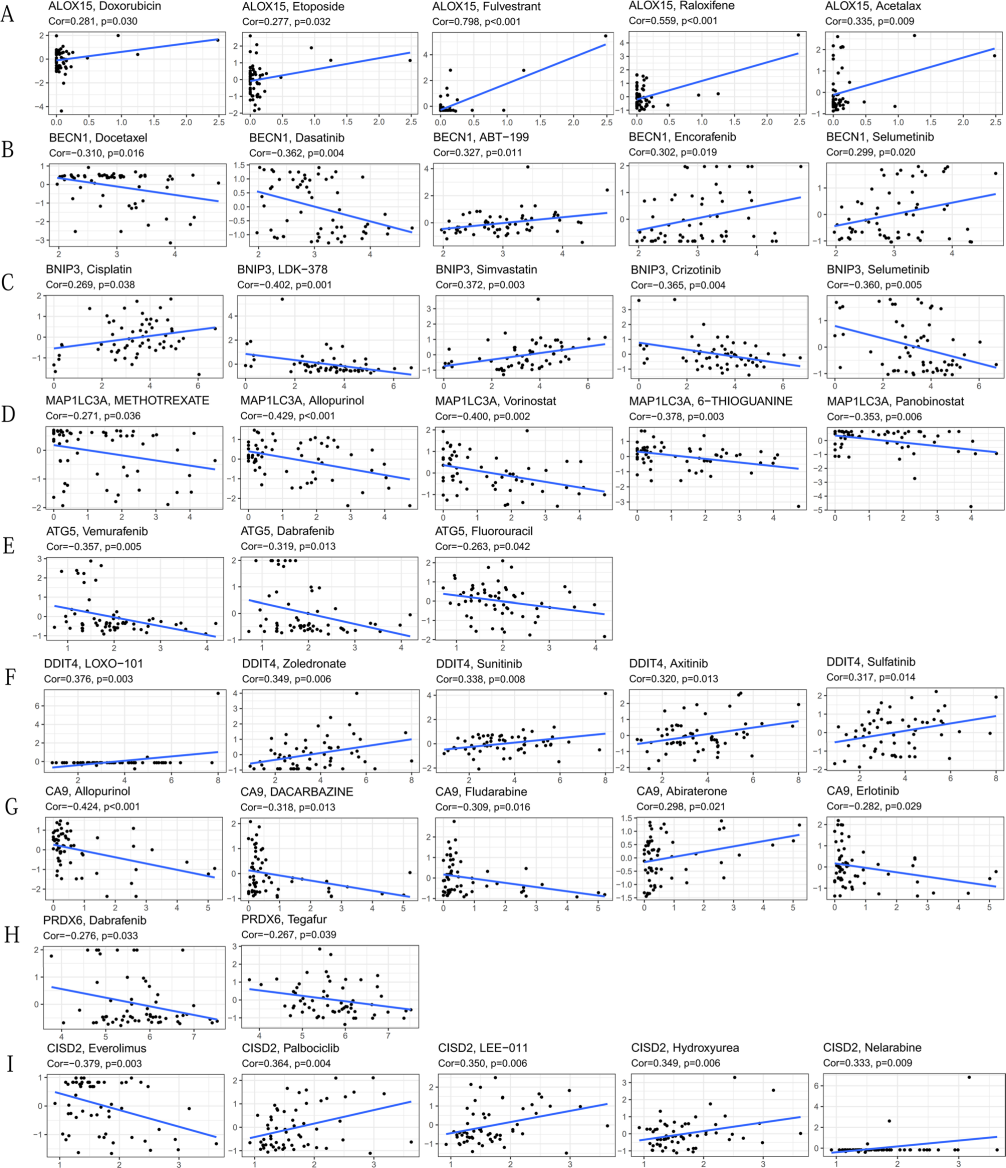
Correlation results of the top 5 drugs with the strongest correlation with 9 PR-DE-FRGs. (**A**) ALOX15. (**B**) BECN1. (**C**) BNIP3. (**D**) MAP1LC3A. (**E**) ATG5. (**F**) DDIT4. (**G**) CA9. (**H**) PRDX6. (**I**) CISD2. The above figures were drawn using R programming language (version 4.0.3, www.r-project.org/).

IPS was used to evaluate the effect of ICIs on sample treatment, and the evaluation value of IPS was positively correlated with the therapeutic effect of ICIs^24^. We analyzed the correlation between IPS and the expression of 9 PR-DE-FRGs. We found that IPS-CTLA4-pos + PD1-pos was negatively correlated with the expression of PRDX6, MAP1LC3A, and ALOX15; and IPS-CTLA-neg + PD1- pos was negatively correlated with MAP1LC3A and ALOX15 (Fig. 12E). The expression of other genes did not show a significant correlation with IPS (Fig. 12F). These results indicated that the expression levels of these three genes could predict the efficacy of ICIs. We also analyzed the correlation between drug sensitivity and the expression of 9 PR-DE-FRGs. We found that the expression level of all PR-DE-FRGs was significantly related to the sensitivity of certain chemotherapeutic drugs (Fig. 13). The figure showed the results of the top 5 drugs with the strongest correlation with the 9 PR-DE-FRGs. In particular, these 9 PR-DE-FRGs and some of the drugs recommended in the latest NCCN treatment of OSCC guidelines also showed a significant correlation, such as the positive correlation between ALOX15 expression and the sensitivity of Doxorubicin and Etoposide (Fig. 13A), negative correlation between BECN1 expression and the sensitivity of Docetaxel (Fig. 13B), positive correlation between BNIP3 expression and the sensitivity of Cisplatin (Fig. 13C) and negative correlation between MAP1LC3A expression and the sensitivity of Methotrexate (Fig. 13D).

### The nomogram can accurately predicted the OSCC patients' survival probability

**Figure 14:**
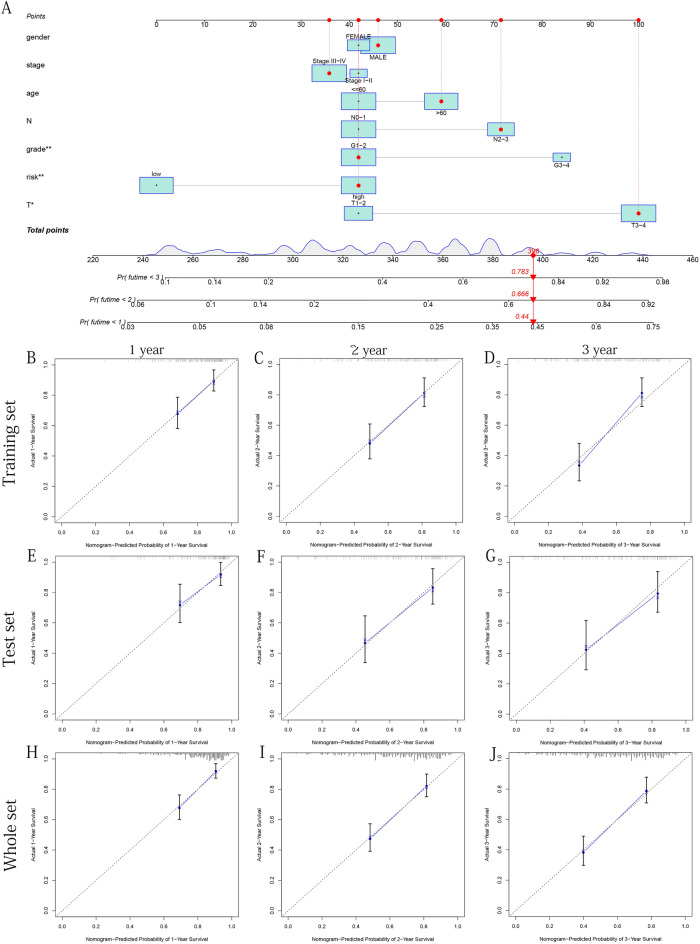
Construct the Nomogram and verify it using internal calibration curves of 1, 2, and 3 years. (**A**) Based on different clinical risk factors and risk scores, the Nomogram is constructed to predict the OS of OSCC patients. (**B-D**) Based on the training set respectively. (**E**–**G**) Based on the test set respectively. (**H**–**J**) Based on the whole set respectively. The above figures were drawn using R programming language (version 4.0.3, www.r-project.org/).

**Figure 15:**
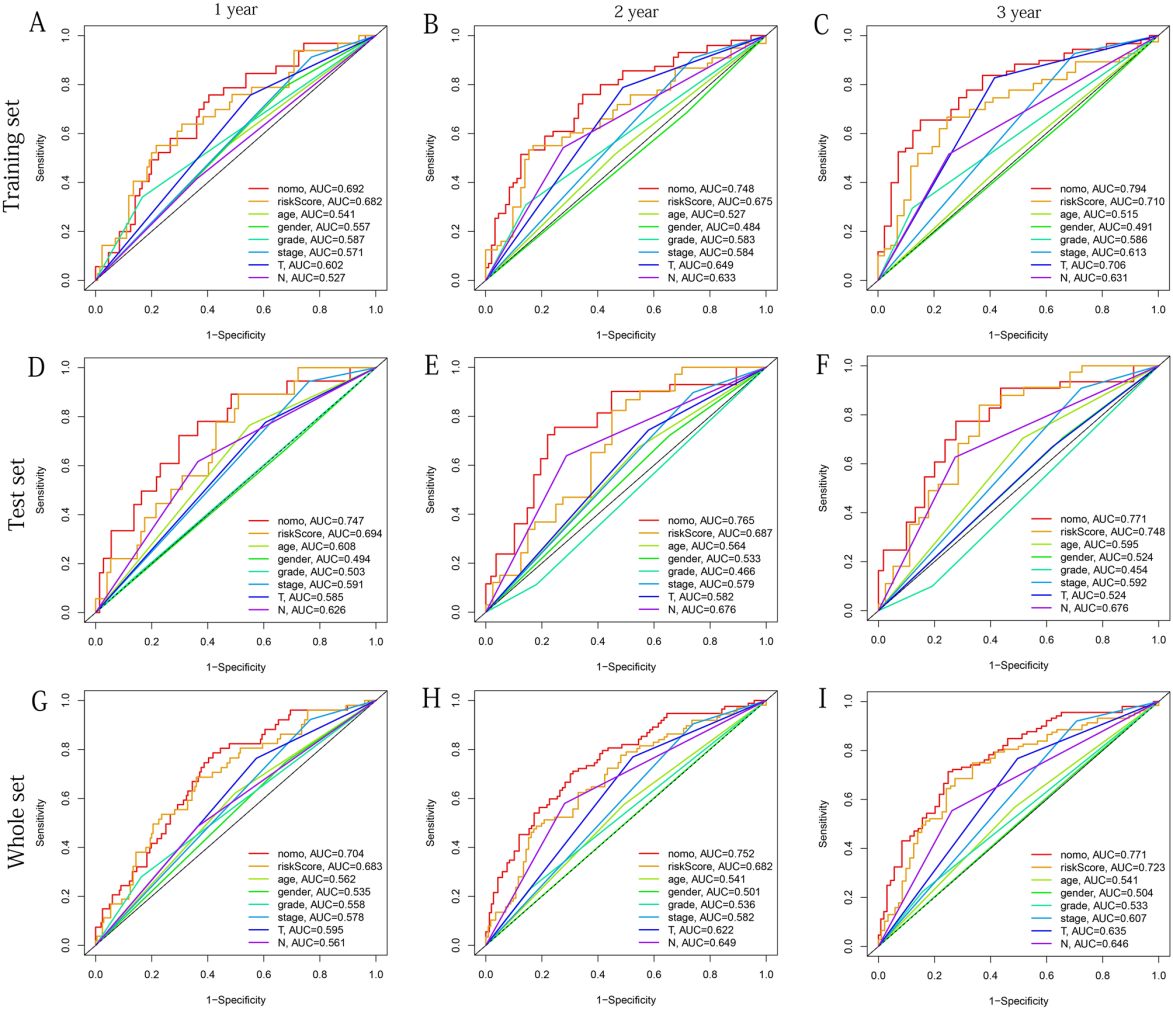
Multivariate ROC curves at 1, 2 and 3 years are used to verify the best predictive ability of Nomogram. (**A**–**C**) Based on the training set, respectively. (**D**–**F**) Based on the test set, respectively. (**G**–**I**) Based on the whole set, respectively. The above figures were drawn using R programming language (version 4.0.3, www.r-project.org/).

To further predict the prognosis of OSCC patients, we established a Nomogram based on the seven factors of age, gender, grade, stage, T, N, and risk group to predict the OS (at 1, 2, and 3-years) (Fig. 14A). We confirmed that the predicted survival rate of Nomogram in the training, test, and whole sets (training set: Fig. 14B–D, test set Fig. 14E–G, whole set: Fig. 14H–J) and the actual 1, 2, and 3-years OS rates had good consistency, suggesting that it had a practical predictive ability for OSCC. To further verified this adequate predictive power, we conducted ROC curves based on three datasets. The results showed that our Nomogram had a brilliant predictive value (AUC value > 0.65) and had a tremendous predictive value for 1, 2, and 3 years of OS. Its predictive ability was better than other clinical factors (training set: Fig. 15A–C, test set: Fig. 15D–F, whole set: Fig. 15G–I).

## Discussion

Nowadays, some studies discussing the influence of FRGs expression on the occurrence of ferroptosis and in-depth analysis of the relationship between ferroptosis and the clinical characteristics of cancer have conducted a systematic analysis of ferroptosis and FRGs. Those results demonstrated that the promising potential of ferroptosis for cancer treatment^26^. For example, Liu et al. implied that the identified ferroptosis-related genes were significantly correlated with glioma progression^27^. Mou et al. demonstrated the expression level of FRGs was significantly associated with the OS of clear cell renal cell carcinoma^28^. Similar results have been reported in lung adenocarcinoma^29,30^. We checked the literature of the PubMed database and found extraordinarily rare studies using FRGs to predict the prognosis of OSCC patients and only involve the TCGA database. There were scarce discussions on the mechanism of FRGs affecting the occurrence, development, and prognosis of OSCC. So, it is worthy of insight. In this study, the data of the five cohorts obtained by the crossover of TCGA, GEO, ICGC and FerrDb database were collated, and 13 PR-DE-FRGs were indicated through the Venn diagram and Univariate COX regression analysis. Further LASSO regression was performed on the training set to obtain nine highly correlated PR-DE-FRGs to construct a prognostic model with superior performance. To further improve the credibility of the conclusion, we used three databases to create six datasets to verify the results. The prognostic analysis results all showed a significant negative correlation between risk score and OS. In-depth Univariate and Multivariate Cox Regression Analyses found that risk score was the best independent predictor of OS. The six sets' ROC curves subsequently confirmed our results, indicating that the model has the best predictive effect. In the enrichment analysis, the high-risk group is enriched for many P53-related biological processes, while the low-risk group is enriched for a large number of immune-related cellular components, molecular functions, and biological processes. It is based on these enrichment results as a guide that we further conduct TME, TP53 Mutation analysis. And the final results showed that TME, Mutation analysis, and cell stemness were all linked to risk scores.

It is worth mentioning that He et al. have identified the ferroptosis-related gene signature in HNSCC, which coincides with our research^31^. But compared to this study, our study shows many advantages. First, the study by He et al. only used the TCGA HNSCC dataset for analysis, while we used both TCGA and GSE30784 cohorts to screen common DE-FRGs. In the performance verification of the model, compared with the only external verification cohort (E-MTAB-8588 cohort) in the study of He et al., our research also used the GSE41613, GSE65858 and ICGC-ORCA-IN cohorts to externally verify the excellent performance of the model. These all reflect the higher accuracy and stability of our results. In the clinical application part, the study by He et al. only explored the guiding value of the model in chemotherapy, while our study also explored the guiding value of the model in immunotherapy. In the part of immune infiltration, He et al.'s research only explored the relationship between 22 kinds of immune cells and the model. Our study also explored the relationship between the model and 13 immune functions. In addition, the correlation analysis of mutation signature and stem cell signature is also not available in the study of He et al.

Univariate Cox analysis of the 9 PR-DE-FRGs used to construct the prognostic model revealed eight genes (CISD2, DDIT4, CA9, ALOX15, ATG5, BECN1, BNIP3, and PRDX6) were identified as prognostic risk factors, and MAP1LC3A was identified as prognostic protective factors. Similarly, the Kaplan–Meier test found that except for MAP1LC3A, the low expression of the other eight genes was significantly related to better OS. Therefore, the prognostic model constructed using these 9 PR-DE-FRGs has a solid ability to predict the prognosis of OSCC patients. Ferroptosis is a type of iron-dependent and lipid peroxidation-dependent nonapoptotic cancer cell death and is closely related to glutathione metabolism^11^. These 9 PR-DE-FRGs can be roughly divided into four categories: (Anti-)Oxidant Metabolism (CISD2, CA9, ALOX15, BNIP3, PRDX6, MAP1LC3A), Glutathione metabolism (BECN1), iron metabolism (CISD2, ATG5), p53-Mediated Regulation (DDIT4)^32^. CISD2 inhibition overcomes head and neck cancer resistance to ferroptotic cell death induced by sulfasalazine via increased accumulation of mitochondrial ferrous iron and lipid reactive oxygen species (ROS)^33^. Cheng et al. implied that Up-regulation of DDIT4 predicts poor prognosis in acute myeloid leukaemia^34^. DDIT4 (also known as REDD1) regulates p53/TP53 mediator apoptosis in response to DNA damage and regulates cell growth, proliferation and survival by inhibiting the activity of mammalian target of rapamycin complex 1 (mTORC1)^35–37^. Previous literature has revealed that DDIT4 expression was much higher in nasopharyngeal carcinoma (NPC) and HNSCC tissues than in adjacent tissues^38,39^. Zhao et al. found that DDIT4 overexpression promotes the proliferation, migration, invasion and inhibits the apoptosis of NPC cells by partially activating the mTOR signaling pathway^39^. Song et al. associated increased tumor level of CA9 mRNA or protein with shorter survival times of patients with pancreatic, kidney, or lung cancers^40^. CA9 plays a role in equilibrating among hypoxia, iron metabolism and redox regulation in Malignant mesothelioma cells^41^. ALOX15 is one of the key genes that cause ferroptosis^42^, by mediating the production of Lipid ROS in various types of tissues and tumors^43^. Both ATG5 and BECN1 are components of the autophagy machinery contribute to ferroptotic cell death^44^. Previous studies revealed that, knockdown or knockout of ATG5 inhibited the BAY-stimulated autophagosome formation, and limited erastin-induced ferroptosis with decreased intracellular ferrous iron levels, and increase cellular ROS^45–47^. BECN1 can promote ferroptosis through the regulation of activity of the cysteine and glutamate antiporter system xc^–^ in cancer cells^48^. Cells transfected with BNIP3 exhibit yielding a morphotype that is typical of necrosis accompanied by rapid and profound mitochondrial dysfunction, one of them characterized by increased reactive oxygen species production^49^. Previous studies have confirmed that PRDX6 can reduce peroxidized cell membrane phospholipids and plays a major role in repairing peroxidized cell membranes. The knockdown of intracellular PRDX6 significantly enhances LOOH and ferroptotic cell death triggered by ferroptosis inducers (Erastin and RSL-3)^50,51^. Giatromanolaki et al. found that cytoplasmic LC3A (also known as MAP1LC3A) expression was the autophagy-related parameter strongly and independently linked with poor prognosis in gastric cancer^52^. Due to the limitations of the resource, we did not conduct further basic experiments for verification, so we verified the correctness of the gene differential expression analysis by obtaining immunohistochemical staining images of PR-DE-FRGs. Stem cell characteristics reveal new drug targets for anti-cancer therapy^23^. Cell stemness usually show high levels of iron in the cells^53^. Our OSCC stem cell characteristics analysis found that CSCs score and risk score showed a significant positive correlation. In the high-risk group, stage T4 and N3 groups had significantly higher mDNAsi. This is consistent with our current knowledge. This conclusion indicated that cancerous cells in our high-risk group are more aggressive and can invade surrounding tissues, which is also reflected in the study of glioblastoma^55^.

The above was primarily the study of 9 PR-DE-FRGs in other diseases. To deeply analyze the biological processes and pathways that these 9 genes may participate in OSCC, we conducted GSEA. Interestingly, the high-risk group was enriched in many P53-related biological processes. And our research shows that the most frequently mutated gene in both the high-risk and low-risk groups is the TP53 gene. Naturally, we further conducted an analysis related to TP53. TP53 was related to the occurrence, development, and prognosis of a variety of cancers, such as metastatic head and neck cancer^56^, lung squamous cell carcinoma^57^, breast cancer^58^, bone and soft tissue sarcomas^59^. P53 suppresses tumor mutations by destroying cells and preventing the proliferation of abnormal genes after DNA damage, but mutated P53 will lose its function^60^. In addition, recent studies have also found that P53 can also exert its tumor suppressor function by inducing apoptosis and ferroptosis^19^. These findings explain the worse OS of patients in the TP53 mutation group, which may be because the TP53 mutation inhibits ferroptosis, which promotes the progression of OSCC and leads to a worse prognosis for patients. Consistent with the findings of Lyu et al., we observed that the content of CD8 T cells were higher in the wild TP53 group compared to mutated TP53^61^. Recent studies have implied that CD8 T cells activated by immunotherapy can promote tumor cell lipid peroxidation and ferroptosis by releasing interferon-gamma (IFNγ), thereby enhancing the anti-tumor efficacy of immunotherapy^13^. These findings explain the worse OS of patients in the TP53 mutation group, which may be because the TP53 mutation inhibits ferroptosis, which promotes the progression of OSCC and leads to a worse prognosis for patients. The low-risk group was enriched in a large number of immune-related cell components, molecular function, and biological process. Therefore, we further analyzed the OSCC immune microenvironment. The results showed that whether the immune score or stromal score, the score of the low-risk group was higher. In addition to aDCs, NK cells, and Th1 cells, the enrichment of 13 other immune cells in the low-risk group was significantly higher than that in the high-risk group. First, higher T-cell co stimulation score was observed in the low-risk group. Previous works of literature have proven that HNSCC patients with high CD8+ T cell penetration have a better prognosis^62^. And a study has shown a significant correlation between increased tumor penetration of CD8+ T cells and their tissue localization in OSCC^63^. Wang et al. also found that CD8+ T cells activated by immunotherapy can make tumors sensitive to ferroptosis by promoting tumor cell lipid peroxidation and IFN+^13^. The beneficial effect of numerous CD20+ B cells in the metastatic lymph nodes of HNSCC^64^. Dendritic cells (DC) play a crucial role in anti-tumor immunity, indicating a good prognosis in OSCC^65^. The high density of tumor-associated macrophages seems to have a better survival rate in colorectal cancer^66^. Tumor-associated mast cells are associated with a good prognosis in esophageal squamous cell carcinoma^67^, ovarian cancer^68^ and diffuse large B-cell lymphoma^69^. In ovarian cancer, the accumulation of tumor-infiltrating lymphocytes (TIL) is a good prognostic factor and can improve its OS^70^. And neutrophils induce iron-dependent accumulation of lipid peroxide in tumor cells by transferring bone marrow-containing oxidase particles into tumor cells, leading to ferroptosis^71^. Combined with the immune cell changes related to the TP53 mutation above, we were surprised to find that CD8 T cells infiltrated higher in the TP53 wild group, indicating that TP53 mutations may inhibit the proliferation of CD8 T cells and cause their content in OSCC to decrease. In the low-risk group, the Type II IFN Response score is higher, which in turn implies that it may be because of the reduction of CD8 T cells that the interferon-gamma (IFNγ) response is also reduced, thereby inhibiting the occurrence of ferroptosis, and ultimately leading to OSCC has a worse prognosis. Besides, low-risk group had higher fractions of CCR, Check-point, HLA, T cell co-stimulation, T cell co-inhibition, and Type II IFN Response scores. These results suggest that the poor prognosis of patients in the high-risk group is likely to be closely related to decreased anti-tumor immunity. Further KEGG pathway analysis showed numerous ferroptosis-related pathways that further revealed that the occurrence, development, and prognosis of OSCC are related to ferroptosis.

They were based on exploring the correlation between ferroptosis and cancer immunotherapy and adjuvant therapy and analyzing whether the prognostic model can predict its efficacy. The results of IPS demonstrated that the expression levels of three genes in nine PR-DE-FRGs could predict the efficacy of ICIs. In particular, the drugs susceptibility analysis showed that all genes significantly correlate with the latest NCCN recommended drugs. Studies have shown that m6A is involved in the regulation of some tumor-targeted therapeutic genes^72^. The results of our research on M6A-related genes again suggest the prospect of the prognostic model in clinical treatment applications. The above results demonstrated that our model could predict the efficacy of immunotherapy and chemotherapy to minimize patients' adverse reactions. Providing accurate and reliable prediction methods for the prognosis of cancer patients to optimize clinical treatment strategies and personalized treatment is the direction that many scholars have been working hard^73^. Therefore, we established a Nomogram based on seven factors to predict the OS of OSCC patients at 1, 2, and 3 years and verified it through various methods. The results implied that our Nomogram has good performance.

In this study, we constructed a model with a good predictive capability based on 9 PR-DE-FRGs and used six datasets to verify its performance in multifaceted directions. And we also discussed the role of ferroptosis in the initiation, progression and prognosis of OSCC from multiple perspectives such as immunity, mutation, stem cell characteristics and clinical application. But there were still some limitations. First of all, this study has not yet performed qRT-PCR to verify the differential expression of 9 PR-DE-FRGs from the RNA transcript levels. Secondly, the immunohistochemical image had 5 PR-DE-FRGs differentially expressed at the protein expression level that has not been verified. Due to limited data sources, we also failed to account for the interference of treatments on the estimated prognostic performance of our prognostic model. And due to various limitations, in reality, it is difficult for us to combine clinical specimens and experiments to verify the relevant conclusions of the mechanism reasoning and clinical application sections. Finally, some conclusions obtained by this study were only reasonable inferences based on the previous research conclusions and our results and only provided a novel perspective for future research.

## Supplementary Information

Supplementary Information 1.

Supplementary Information 2.

Supplementary Information 3.

## Data Availability

The datasets analyzed in this study came from databases shared publicly. Data can be obtained from: CellMinerdiscover.nci.nih.gov/cellminer, FerrDbwww.zhounan.org/ferrdb, GEOwww.ncbi.nlm.nih.gov/geo/, ICGCdcc.icgc.org/releases/current/Projects/ORCA-IN, TCGAcancergenome.nih.gov/, TCIAtcia.at/home,the human protein atlas databasewww.proteinatlas.org/.
