# Supplementary material for: A ferroptosis-related prognostic model with excellent clinical performance based on the exploration of the mechanism of oral squamous cell carcinoma progression
Source: Sci Rep. 2023 Jan 26;13:1461. doi: 10.1038/s41598-023-27676-3 (PMC9880000; doi:10.1038/s41598-023-27676-3)
Supplement: Supplementary file 1 — Supplementary Information 1. [file 41598_2023_27676_MOESM1_ESM.doc]

Supplementary Figure legend


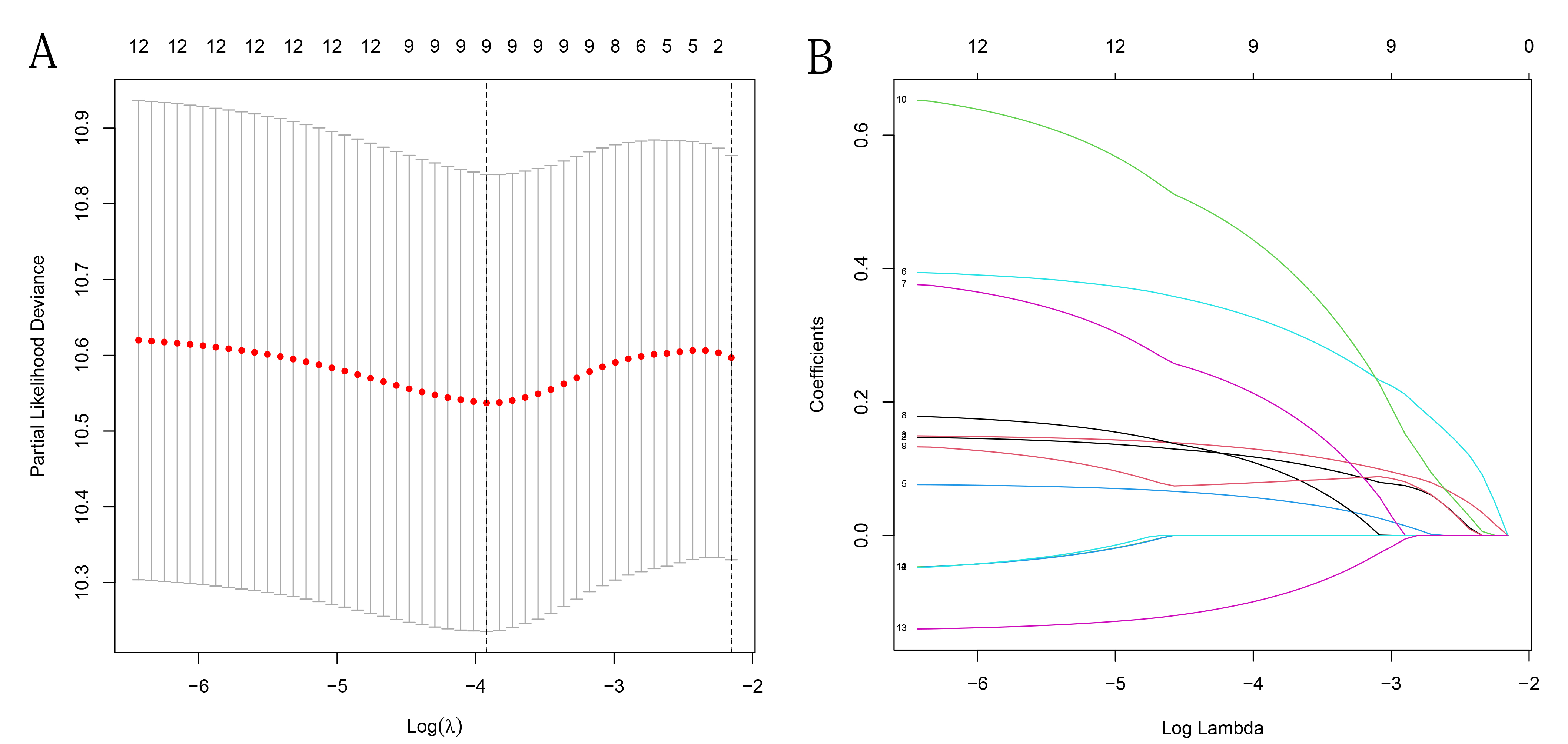


Supplementary Figure S1. (A-B) Based on the training set, LASSO regression is used to obtain the minimum standards and coefficients. The above figures were drawn using R programming language (version 4.0.3, www.r-project.org/).


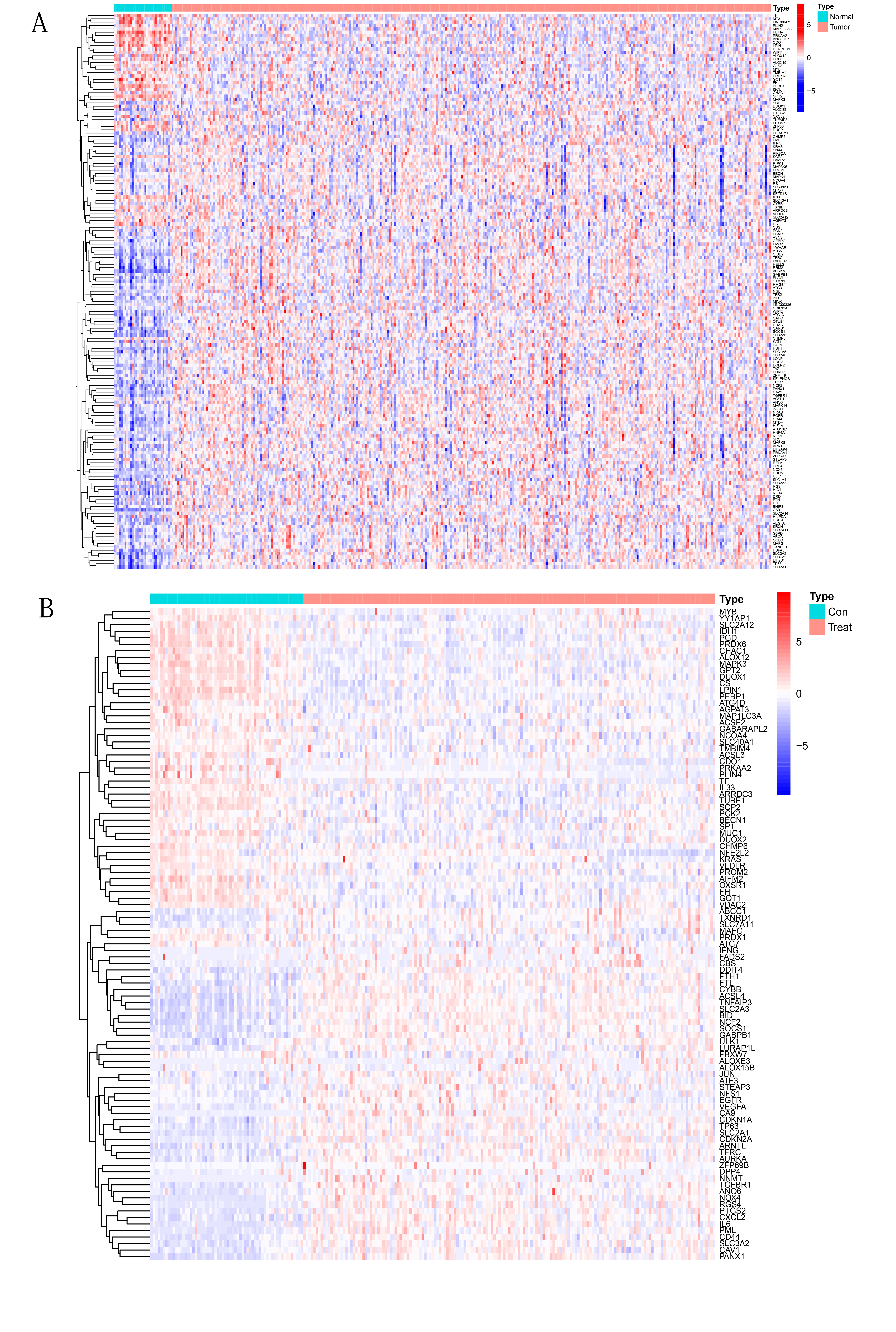


Supplementary Figure S2. Heat maps based on DE-FRGs of TCGA and GSE30784 cohorts. (A) TCGA cohort. (B) GSE30784 cohort. The above figures were drawn using R programming language (version 4.0.3, www.r-project.org/).


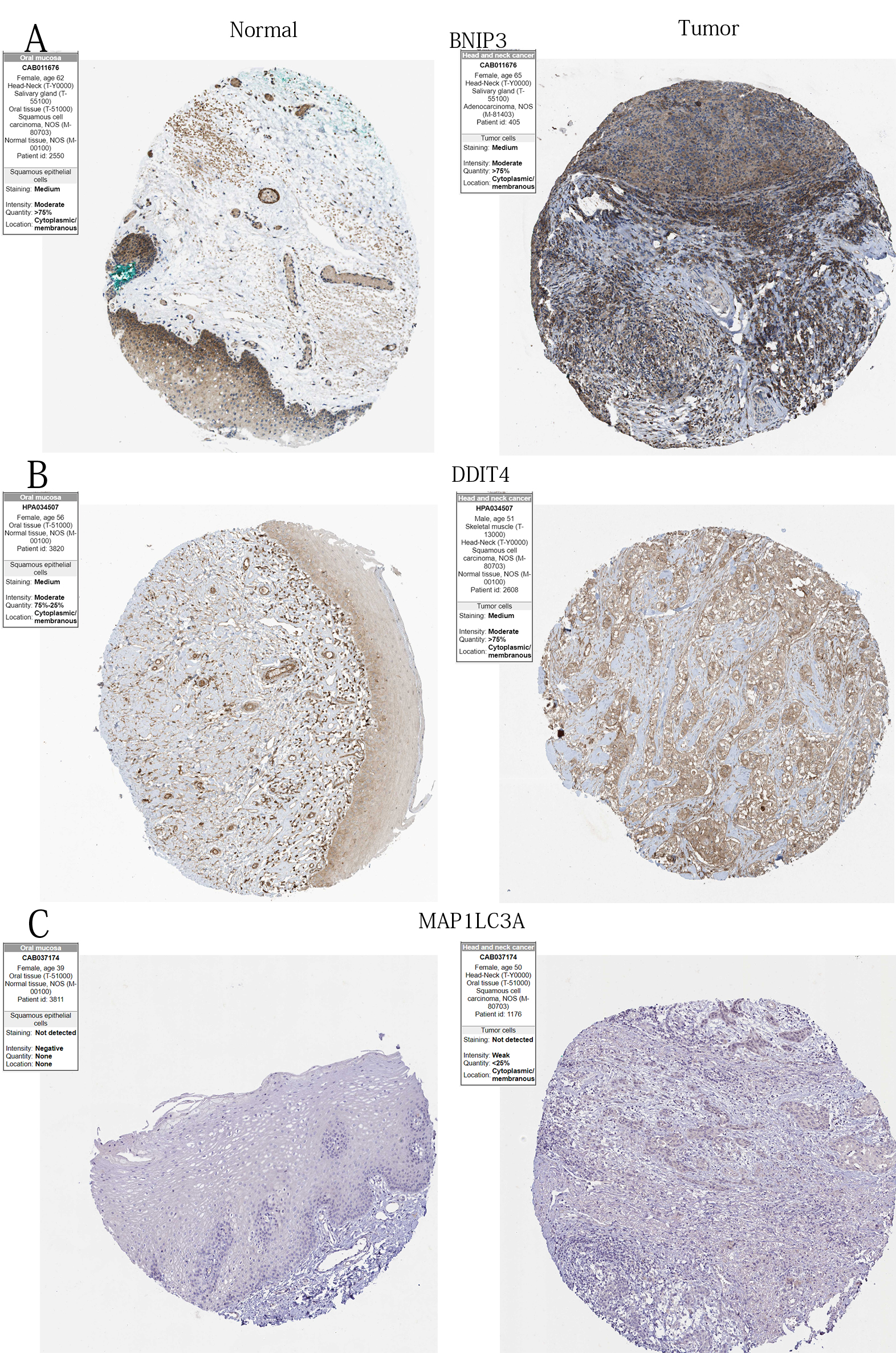


Supplementary Figure S3. IHC images based on the expression of MAP1LC3A, BNIP3 and DDIT4 in OSCC and normal oral tissues. (A) BNIP3. (B) DDIT4. (C) MAP1LC3A.


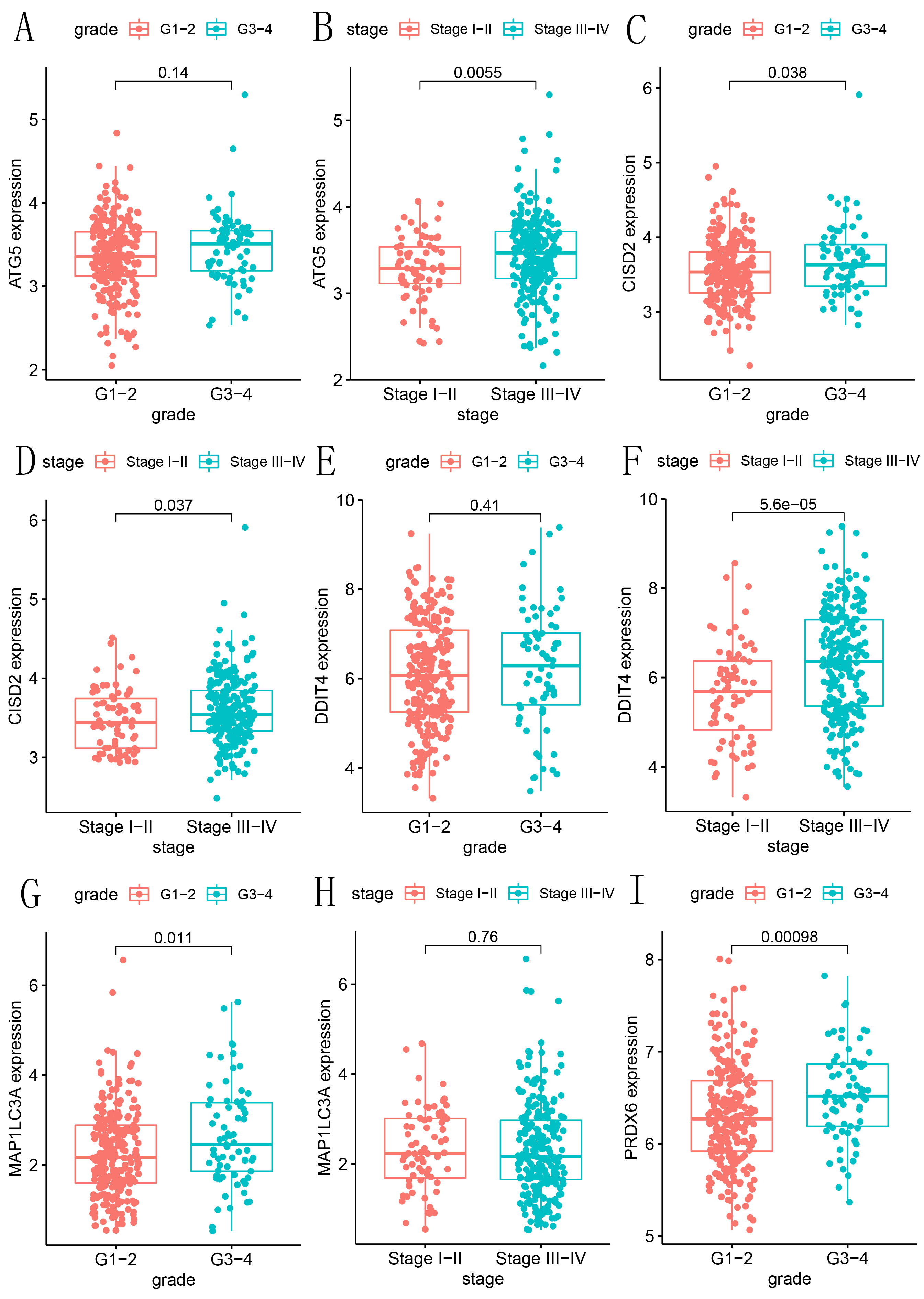


Supplementary Figure S4. The expression differences of ATG5, CISD2, DDIT4, MAP1LC3A and PRDX6 between subgroups with different clinicopathological features. (A-B) ATG5. (C-D) CISD2. (E-F) DDIT4. (G-H) MAP1LC3A. (I) PRDX6. The above figures were drawn using R programming language (version 4.0.3, www.r-project.org/).


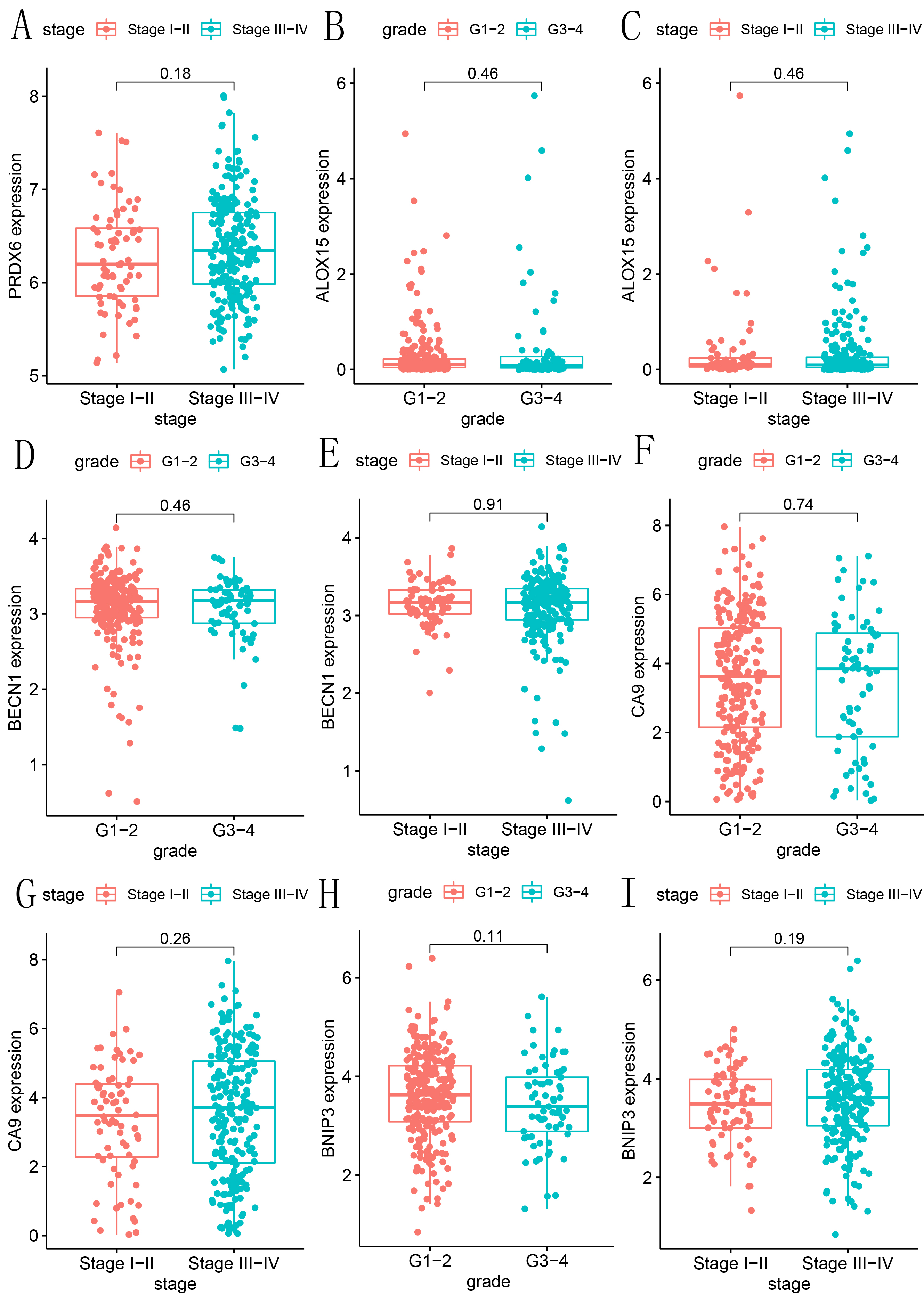


Supplementary Figure S5. The expression differences of PRDX6, ALOX15, BECN1, CA9 and BNIP3 between subgroups with different clinicopathological features. (A) PRDX6. (B-C) ALOX15. (D-E) BECN1. (F-G) CA9. (H-I) BNIP3. The above figures were drawn using R programming language (version 4.0.3, www.r-project.org/).
