# Supplementary material for: A ferroptosis-related prognostic model with excellent clinical performance based on the exploration of the mechanism of oral squamous cell carcinoma progression
Source: Sci Rep. 2023 Jan 26;13:1461. doi: 10.1038/s41598-023-27676-3 (PMC9880000; doi:10.1038/s41598-023-27676-3)
Supplement: Supplementary file 2 — Supplementary Information 2. [file 41598_2023_27676_MOESM2_ESM.docx]

**Supplementary Table 1. The DE-FRGs used to construct the model and the corresponding coefficients**

| Gene | Coef |
| --- | --- |
| BNIP3 | 0.11544 |
| DDIT4 | 0.12777 |
| CA9 | 0.05508 |
| CISD2 | 0.32123 |
| PRDX6 | 0.20484 |
| ALOX15 | 0.10414 |
| ATG5 | 0.07954 |
| BECN1 | 0.43047 |
| MAP1LC3A | -0.09567 |
