# Supplementary material for: A ferroptosis-related prognostic model with excellent clinical performance based on the exploration of the mechanism of oral squamous cell carcinoma progression
Source: Sci Rep. 2023 Jan 26;13:1461. doi: 10.1038/s41598-023-27676-3 (PMC9880000; doi:10.1038/s41598-023-27676-3)
Supplement: Supplementary file 3 — Supplementary Information 3. [file 41598_2023_27676_MOESM3_ESM.docx]

Supplementary Table 2. Primers for BNIP3, DDIT4 and MAP1LC3A.

| **Gene** | Forward Primer | Reverse Primer |
| --- | --- | --- |
| **β-Actin** | TGGCACCCAGCACAATGAA | CTAAGTCATAGTCCGCCTAGAAGCA |
| **BNIP3** | CAGGGCTCCTGGGTAGAACT | CTACTCCGTCCAGACTCATGC |
| **DDIT4** | TGAGGATGAACACTTGTGTGC | CCAACTGGCTAGGCATCAGC |
| **MAP1LC3A** | AACATGAGCGAGTTGGTCAAG | GCTCGTAGATGTCCGCGAT |
